# Gastrointestinal adverse events of metformin treatment in patients with type 2 diabetes mellitus: A systematic review, meta-analysis and meta-regression of randomized controlled trials

**DOI:** 10.3389/fendo.2022.975912

**Published:** 2022-09-14

**Authors:** Katarzyna Nabrdalik, Karolina Skonieczna-Żydecka, Krzysztof Irlik, Mirela Hendel, Hanna Kwiendacz, Igor Łoniewski, Kornelia Januszkiewicz, Janusz Gumprecht, Gregory Y. H. Lip

**Affiliations:** ^1^ Department of Internal Medicine, Diabetology and Nephrology, Faculty of Medical Sciences in Zabrze, Medical University of Silesia, Katowice, Poland; ^2^ Liverpool Centre for Cardiovascular Science, University of Liverpool, Liverpool John Moores University and Liverpool Heart & Chest Hospital, Liverpool, United Kingdom; ^3^ Department of Biochemical Science, Pomeranian Medical University, Szczecin, Poland; ^4^ Students’ Scientific Association by the Department of Internal Medicine, Diabetology and Nephrology in Zabrze, Faculty of Medical Sciences in Zabrze, Medical University of Silesia, Katowice, Poland; ^5^ Department of Clinical Medicine, Aalborg University, Aalborg, Denmark

**Keywords:** Adverse events, diarrhea, dose, formulation, gastrointestinal, meta-analysis, metformin

## Abstract

**Introduction:**

Metformin is the first choice drug in the treatment of type 2 diabetes mellitus but its administration may be linked to gastrointestinal adverse events limiting its use.

**Objectives:**

The objective of this systematic review and meta-analysis was to assess the risk of gastrointestinal adverse events related to metformin use in patients with type 2 diabetes treated with metformin.

**Methods:**

PUB MED/CINAHL/Web of Science/Scopus were searched from database inception until 08.11.2020 for articles in English and randomized controlled trials related to patients with type 2 diabetes treated with metformin were included.

**Results:**

From 5315 publications, we identified 199 potentially eligible full-text articles. Finally, 71 randomized controlled trials were included in the meta-analysis. In these studies, metformin use was associated with higher risk of abdominal pain, diarrhea and nausea comparing to control. The risks of abdominal pain and nausea were highest comparing to placebo. Bloating risk was only elevated when metformin treatment was compared to DPP4i.

**Conclusions:**

The risk of gastrointestinal adverse events such as abdominal pain, nausea and diarrhea is higher in type 2 diabetes patients treated with metformin compared to other antidiabetic drugs. There is a higher risk of bloating and diarrhea with metformin immediate-release than with metformin extended release formulation.

**Systematic Review Registration:**

https://www.crd.york.ac.uk/prospero/display_record.php?ID=CRD42021289975, identifier CRD42021289975.

## 1 Highlights

Gastrointestinal adverse events of metformin treatment are the most common and are assessed to affect up to 20% of patients. It is commonly advised to increase the metformin starting dose gradually and to use the metformin extended release formulation to avoid gastrointestinal adverse events but this knowledge comes from single studies, not systematic reviews with meta-analyses.

We demonstrate that the risk of abdominal pain, nausea and diarrhea is higher in patients with type 2 diabetes mellitus treated with metformin compared to other antidiabetic drugs or placebo. Metformin immediate-release is associated with a higher risk of diarrhea and bloating compared to metformin extended release.

The impact on clinical practice in the foreseeable future of our observations is that in patients with bloating or diarrhea related to metformin treatment It may be worth to change the formulation of metformin from immediate release to extended release to reduce the severity of these gastrointestinal symptoms.

## 2 Introduction

Metformin is well established as a drug therapy in diabetes. In 2005, the International Diabetes Federation (IDF) recommended metformin as a first-line treatment for type 2 diabetes mellitus (T2DM) and since 2011, the WHO included metformin in its list of essential medicines and it still remained on this list in the year 2022 (1). The ADA/EASD recommendations retain metformin as the starting therapy in patients with a new diagnosis of T2DM (2). In 2019, the European Society of Cardiology (ESC) guidelines on diabetes, pre-diabetes, and cardiovascular disease (CVD) recommended the use of new antidiabetic drugs of proven cardioprotective properties, namely, sodium-glucose cotransporter inhibitor (SGLT2i) or glucagon-like peptide-1 receptor agonist (GLP-1RA) in patients with established atherosclerotic CVD or high/very high cardiovascular (CV) risk as the first-line therapy (3). However, metformin was the baseline therapy in most participants in recently performed cardiovascular outcomes trials with the use of SGLT2i and GLP-1RA and cardiovascular benefits of these drugs remain largely unknown in metformin-naïve individuals since there are no head-to-head comparisons of metformin with these newer agents (4). Moreover, metformin’s beneficial effects on endothelial dysfunction as well as atherosclerotic cardiovascular disease in T2DM have been proven (5, 6).

Tolerability is the key influence of any drug on real-world efficacy and it is detrimental for patients’ quality of life and adherence. Unfortunately, many patients treated with metformin do not tolerate it due to gastrointestinal (GI) adverse events (AEs). Diarrhea, nausea and vomiting are common AEs of metformin, occurring in ~20% of the patients and, in some cases, leading to lower adherence, discontinuation of the treatment and worse health-related quality of life (7–9). The mechanism(s) of the GI intolerance in patients treated with metformin are not fully understood. The proposed hypotheses involve the accumulation of serotonin (10), histamine (11) or bile acids (12), as well as genetic predisposition related to organic cation transporter 1 (*OCT*1) gene polymorphism (13, 14). Metformin intake also influences the gut microbiota composition in men with normal glucose metabolism and that pre-treatment bacterial genera may determine the development of metformin GI adverse effects (15).

There is also limited evidence that gradual up-titration of the metformin starting dose or extended release formulation of metformin may lead to depletion of symptoms of metformin GI intolerance (16). In approximately 5% of individuals treated with metformin, the severity of the GI AEs still leads to treatment discontinuation (8). Even though there are other antidiabetic drugs which can be used when metformin is not tolerated, these are either expensive (eg. newly marketed agents) or may cause hypoglycemia (eg. sulfonylurea), what is especially dangerous in elderly people (17). Additionally, there are not enough high-quality data regarding differences in clinical outcomes (especially with long-term use) or cost-effectiveness of alternatives to metformin to be able to unequivocally support any of them. Robust data on efficacy, safety and low cost of metformin, therefore, highlights its role as first-line therapy in T2DM (17).

Despite the very widespread clinical use of metformin, there is a lack of systematic evidence regarding the risk of GI AEs of the drug compared to other glucose-lowering drugs or placebo with the exception of recent meta-analyses comparing different metformin formulations (18–20) and network meta-analyses that focused mainly on drugs other than metformin (21, 22). Our aim was to assess the risk of GI AEs of metformin treatment in T2DM patients through performing a systematic review and meta-analysis with meta-regression of randomized controlled trials (RCTs).

## 3 Methods

The protocol for this systematic review, metanalysis and meta-regression has been registered on the International prospective register of systematic reviews (Prospero database registration no. CRD42021289975). For this purpose, we followed the Preferred Reporting Items for Systematic Reviews and Meta-analysis (PRISMA) statement (23). Search strategy, inclusion and exclusion criteria are available in the Electronic Supplementary Material (ESM).

### 3.1 Data and resource availability

Search strategy, inclusion and exclusion criteria are available in the Electronic Supplementary Material (ESM). More structured database with the entirety of extracted data is available on request.

### 3.2 Study selection

We included RCTs (including active and placebo or any other antidiabetic drugs control arms either alone or in combination with metformin) related to T2DM patients treated with metformin. We investigated metformin (any dose alone or in combination with other anti-diabetic medications – MET + add-on) as intervention for any health outcome in T2DM patients. For interventions, we considered separately comparisons of metformin vs placebo or vs active controls.

The main outcome was to assess the risk of gastrointestinal AEs related to metformin treatment in T2DM patients. The following GI AEs related to metformin use were assessed: abdominal pain, bloating, constipation, diarrhea, nausea and vomiting as well as the risk of discontinuation of therapy due to AEs.

Two authors (KI, MH) performed the primary screening independently (i.e. title/abstract screening). When eligibility selection differed, the final decision was taken after consensus with a clinical leader (KN). The full-text screening was performed by two authors independently (KI, MH). Zotero reference manager was used for deduplication of results.

### 3.3 Data extraction

Two reviewers (KI and MH) independently extracted data on the study design, country, sponsorship, and aims of the study first. We characterized study group by participants’ age, sex, BMI and ethnicity along with data on comorbidities. We looked at the type of metformin used (immediately released – IR, extended release – XR and delayed release - DR), its dose and duration of the drug intervention. Data on metformin treatment prior to randomization was collected. This corresponded to earlier metformin treatment, considered as an inclusion criterion of the trial or run-in period with metformin therapy. Comparators were grouped according to their similar mechanism of action (the same class of medication) by clinical leader (KN) as demonstrated in **Table 1**. Additionally, a comparison of different metformin formulations (metformin immediate release or metformin extended or delayed release) or metformin with a combination of metformin and other antidiabetic drug was classified into separate subgroup (MET/MET + add on).

**Table 1:** Grouping of comparators in the present study.

**SGLT2i**
Empagliflozin
Dapagliflozin
Canagliflozin
**GLP-1RA**
Liraglutide
Dulaglutide
Exenatide
**DPP4i**
Sitagliptin
Vildagliptin
Saxagliptin
Linagliptin
Vildagliptin
Saxagliptin
Gemigliptin
Alogliptin
**SULFONYLUREA DERIVATIVES**
Glyburide
Glipizide
Glimepiride
**PPARγ RECEPTOR AGONISTS**
Pioglitazone
Rosiglitazone
**GLINIDES and α-GLUCOSIDASE INHIBITOR (OTHER)**
Repaglinide
Acarbose

DPP4i, dipeptidyl peptidase-4 inhibitor; SGLT2i, sodium-glucose co-transporter-2 inhibitor; GLP-1RA, glucagonlike peptide-1 receptor agonist; PPARγ, peroxisome proliferator- activated receptor gamma.

If any of the collected data were not reported in the published article, they were extracted from the trial registry (ClinicalTrials.gov) whenever possible. Inconsistencies were resolved by consensus, with a clinical leader (KN) being involved. Decisions made during data extraction process, that did not result from inclusion and exclusion criteria are described in ESM.

### 3.4 Outcomes

Co-primary outcomes were the risk of: i) abdominal pain, ii) bloating, iii) constipation, iv) diarrhea, v) nausea, vi) vomiting. The secondary outcome was the risk of therapy discontinuation due to AEs.

### 3.5 Risk of bias assessment

Two reviewers (HK and KJ) independently assessed the risk of bias using the Cochrane Collaboration’s tool for assessing the risk of bias. When a discrepancy occurred, a third author (IŁ) was involved. The quality of a study was reported as high when there were more than three low risk of bias assessments.

### 3.6 Data synthesis and statistical analysis

We conducted a random effects meta-analysis of outcomes for which ≥2 studies contributed data, using Comprehensive Meta-Analysis V3 (http://www.meta-analysis.com). A subgroup analyses regarding type of comparator (as in **Table 1**) was performed and displayed using forest plots. The between-study variance (τ^2^) was estimated using the method of moments (DerSimonian and Laird) (24) and the assumption of homogeneity in effects was tested using the Q statistic with k-1 degree of freedom (k – the number of studies). For nominal outcomes the summary risk ratio (RR) was calculated. A two-tailed Z test was used to test the null hypothesis that the summary effect is zero. In addition to classical meta-analysis, a meta-regression was performed under the random-effects model for both continuous and nominal study level covariates. The regression models with single covariates were fit. Meta-regression variables included: i) duration of intervention (continuous moderator), ii) dosage of metformin (continuous moderator), iii) type of metformin (IR vs. XR+DR) used (categorical moderator), iv) preexisting metformin treatment (categorical moderator), v) ethnicity of the participants White, Asian or diverse (categorical moderator). Funnel plots were inspected to quantify whether publication bias could have influenced the results. Finally, we inspected funnel plots and used Egger’s regression test and the Duval and Tweedie’s trim and fill method if necessary, to quantify whether publication bias could have influenced the results (25, 26). All analyses were two-tailed with alpha=0.05.

The *post hoc* sensitivity analyses included a meta-regression to investigate the potential influence of the type and dose of metformin on the co-primary outcomes. A subgroup analysis with children study exclusion was applied.

### 3.7 Ethics

The study required no ethics committee approval.

## 4 Results

### 4.1 Search results

**Figure 1:**
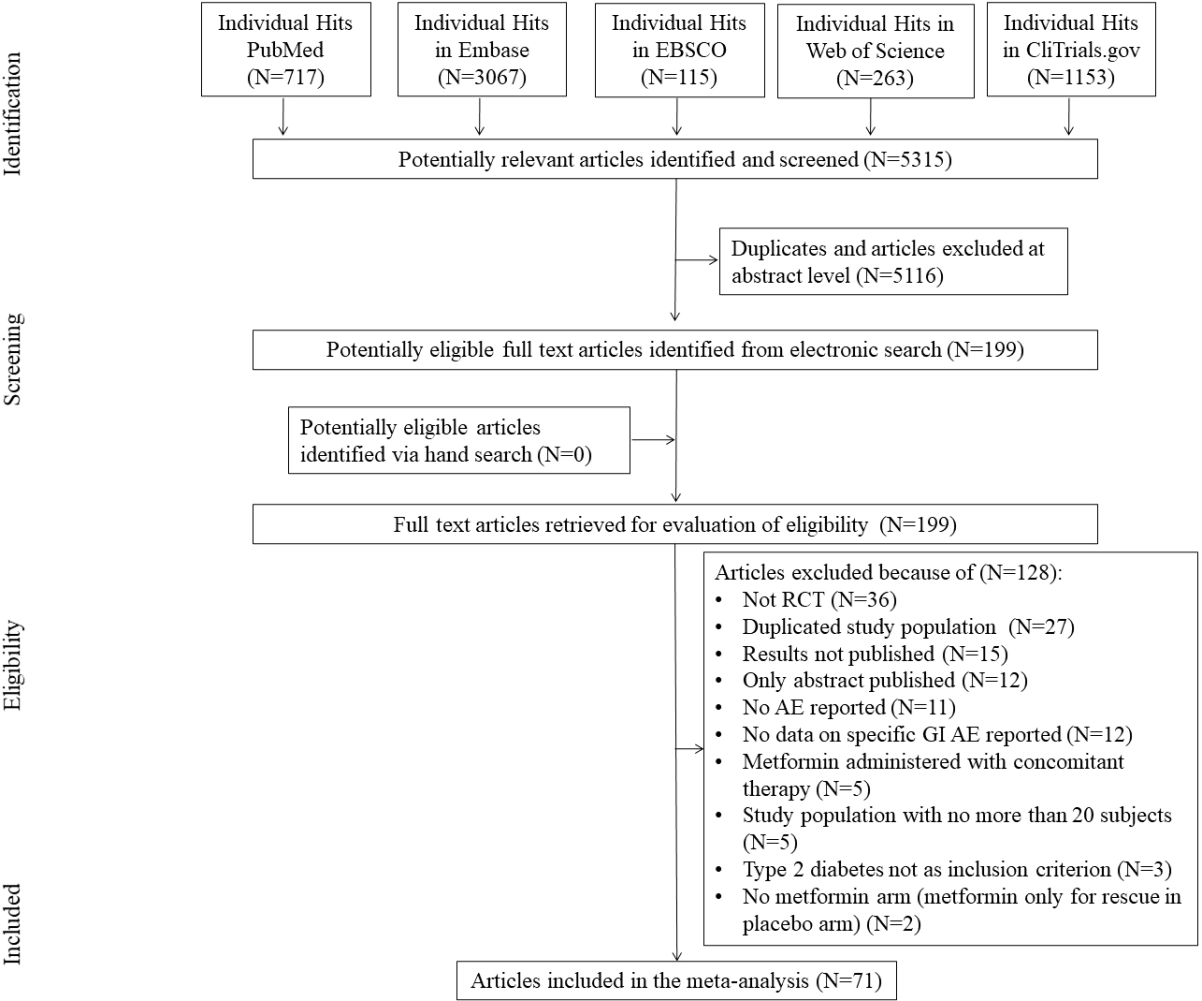
Study flow chart.

The initial search yielded 5315 hits. There were 5116 studies excluded as duplicates and/or after evaluation at the title/abstract level. There were no studies identified *via* hand search. Eventually, 199 full-text articles were reviewed. Of those, 128 were excluded due to not fitting inclusion criteria. Reasons for exclusion are presented in **Figure 1**, yielding 71 studies and 98 arms that were included in the meta-analysis (**Figure 1**).

### 4.2 Study and studied subjects’ characteristics

Altogether, 71 studies (98 arms) including the total number of n=55,042 patients that were randomized to metformin and comparator arms and 54,445 persons analyzed were included into the final synthesis. Studies were mostly conducted in multiple clinical centers and sponsored by industry (n=71 arms, 71%). Predominantly, the analyses were of intention to treat type (n = 94 arms, 94%). There were patients of both sexes included, with the mean presence of males equal to 52.0%. The median age of study participants was 54.0 years (range 13.8-70.9). Typically (n=78 arms, 78%) study patients started the metformin treatment in the trial they took part in and the most common (n=66 arms, 66%) metformin IR was used for a mean time of 179.1 days. Data on other studies and participants’ characteristics are summarized in ESM **Tables S1**–**S3**.

### 4.3 Effect sizes

The proportions of particular GI complications linked to metformin treatment and number of participants in each study has been presented in ESM **Table S4**. We found that the incidence of abdominal pain, bloating, constipation, diarrhea, nausea and vomiting were 6.54, 9.15, 2.27, 12.94, 6.45 and 3.76 percentages respectively. The overall effect sizes for tested outcomes have been presented in **Table 2**.

**Table 2:** The effect sizes of all study outcomes.

Time point	Number of studies	Point estimate	Lower limit	Upper limit	Test Z (z value)	Test Z (p value)	Q value	df(Q)	p value	heterogeneity (I^2^* from fixed effect analysis)
**Abdominal pain**										
DPP4 inhibitor	9	1.773	1.327	2.370	3.872	<0.001	7.754	8.000	0.458	0.000
MET/MET+add on	18	1.202	0.984	1.468	1.800	0.072	14.515	17.000	0.630	0.000
Other	1	1.939	0.185	20.350	0.552	0.581	0.000	0.000	1.000	0.000
PBO	13	1.981	1.294	3.031	3.148	0.002	3.020	12.000	0.995	0.000
PPARγ receptor agonist	2	1.599	0.929	2.752	1.695	0.090	0.205	1.000	0.651	0.000
Sulfonylurea derivatives	7	1.346	1.131	1.603	3.338	0.001	6.793	6.000	0.340	11.672
Total between							7.841	5.000	0.165	
Overall	50	1.491	1.211	1.836	3.768	<0.001	40.127	49.000	0.813	0.000
**Bloating**										
DPP4 inhibitor	4	2.507	1.073	5.857	2.123	0.034	5.794	3.000	0.122	48.221
MET/MET+add on	11	0.780	0.518	1.174	-1.193	0.233	28.689	10.000	0.001	65.143
Other	2	0.573	0.132	2.494	-0.742	0.458	4.224	1.000	0.040	76.323
Sulfonylurea derivatives	1	7.500	0.338	166.221	1.275	0.202	0.000	0.000	1.000	0.000
Total between							8.073	3.000	0.045	
Overall	18	1.266	0.480	3.336	0.476	0.634	49.530	17.000	<0.001	65.677
**Constipation**										
DPP4 inhibitor	5	0.707	0.409	1.225	-1.236	0.216	6.665	4.000	0.155	39.981
GLP-1 receptor agonist	2	0.322	0.096	1.082	-1.833	0.067	1.234	1.000	0.267	18.981
MET/MET+add on	5	0.911	0.442	1.879	-0.251	0.801	2.593	4.000	0.628	0.000
Other	1	0.970	0.135	6.944	-0.031	0.976	0.000	0.000	1.000	0.000
PBO	3	1.357	0.430	4.284	0.520	0.603	2.002	2.000	0.368	0.088
PPARγ receptor agonist	1	1.767	0.431	7.248	0.790	0.429	0.000	0.000	1.000	0.000
Total between							4.542	5	0.474	
Overall	17	0.839	0.489	1.440	-0.638	0.523	17.887	16.000	0.331	10.548
**Diarrhea**										
DPP4 inhibitor	13	2.878	2.209	3.749	7.837	<0.001	31.976	12.000	0.001	62.472
GLP-1 receptor agonist	2	1.919	0.926	3.981	1.752	0.080	0.437	1.000	0.508	0.000
MET/MET+add on	30	1.168	0.972	1.403	1.660	0.097	55.791	29.000	0.002	48.020
Other	3	4.039	1.175	13.887	2.216	0.027	2.044	2.000	0.360	2.148
PBO	26	2.838	2.148	3.749	7.339	<0.001	27.663	25.000	0.324	9.626
PPARγ receptor agonist	6	3.417	2.340	4.990	6.361	<0.001	3.778	5.000	0.582	0.000
SGLT2 inhibitor	5	2.490	1.443	4.297	3.278	0.001	3.049	4.000	0.550	0.000
Sulfonylurea derivatives	7	2.679	1.850	3.878	5.220	<0.001	6.809	6.000	0.339	11.879
Total between							58.248	7	0.0	
Overall	92	2.445	1.656	3.609	4.500	<0.001	263.355	91.000	<0.001	65.446
**Nausea**										
DPP4 inhibitor	10	2.333	1.754	3.103	5.820	<0.001	11.080	9.000	0.270	18.772
GLP-1 receptor agonist	3	1.229	0.826	1.829	1.019	0.308	5.082	2.000	0.079	60.648
MET/MET+add on	26	0.927	0.773	1.113	-0.811	0.417	22.127	25.000	0.628	0.000
Other	1	1.939	0.381	9.861	0.798	0.425	0.000	0.000	1.000	0.000
PBO	23	2.680	1.919	3.744	5.782	<0.001	13.690	22.000	0.912	0.000
PPARγ receptor agonist	3	1.669	1.111	2.506	2.469	0.014	0.071	2.000	0.965	0.000
SGLT2 inhibitor	4	1.568	0.785	3.131	1.274	0.203	4.975	3.000	0.174	39.695
Sulfonylurea derivatives	6	1.676	1.365	2.058	4.930	<0.001	2.682	5.000	0.749	0.000
Total between							49.871	7.000	0.000	
Overall	76	1.641	1.169	2.302	2.866	0.004	109.578	75.000	0.006	31.555
**Vomiting**										
DPP4 inhibitor	7	1.934	1.224	3.057	2.825	0.005	6.375	6.000	0.383	5.876
GLP-1 receptor agonist	3	0.763	0.409	1.422	-0.852	0.394	1.435	2.000	0.488	0.000
MET/MET+add on	18	0.730	0.494	1.079	-1.580	0.114	13.488	17.000	0.703	0.000
PBO	10	3.281	1.893	5.684	4.237	<0.001	7.295	9.000	0.606	0.000
PPARγ receptor agonist	1	1.588	0.819	3.080	1.370	0.171	0.000	0.000	1.000	0.000
SGLT2 inhibitor	1	7.000	0.868	56.455	1.827	0.068	0.000	0.000	1.000	0.000
Sulfonylurea derivatives	5	1.717	1.309	2.250	3.911	<0.001	2.310	4.000	0.679	0.000
Total between							29.629	6.000	0.000	
Overall	45	1.554	0.970	2.489	1.834	0.067	60.531	44.000	0.050	27.310

#### 4.3.1 Abdominal pain risk

**Figure 2:**
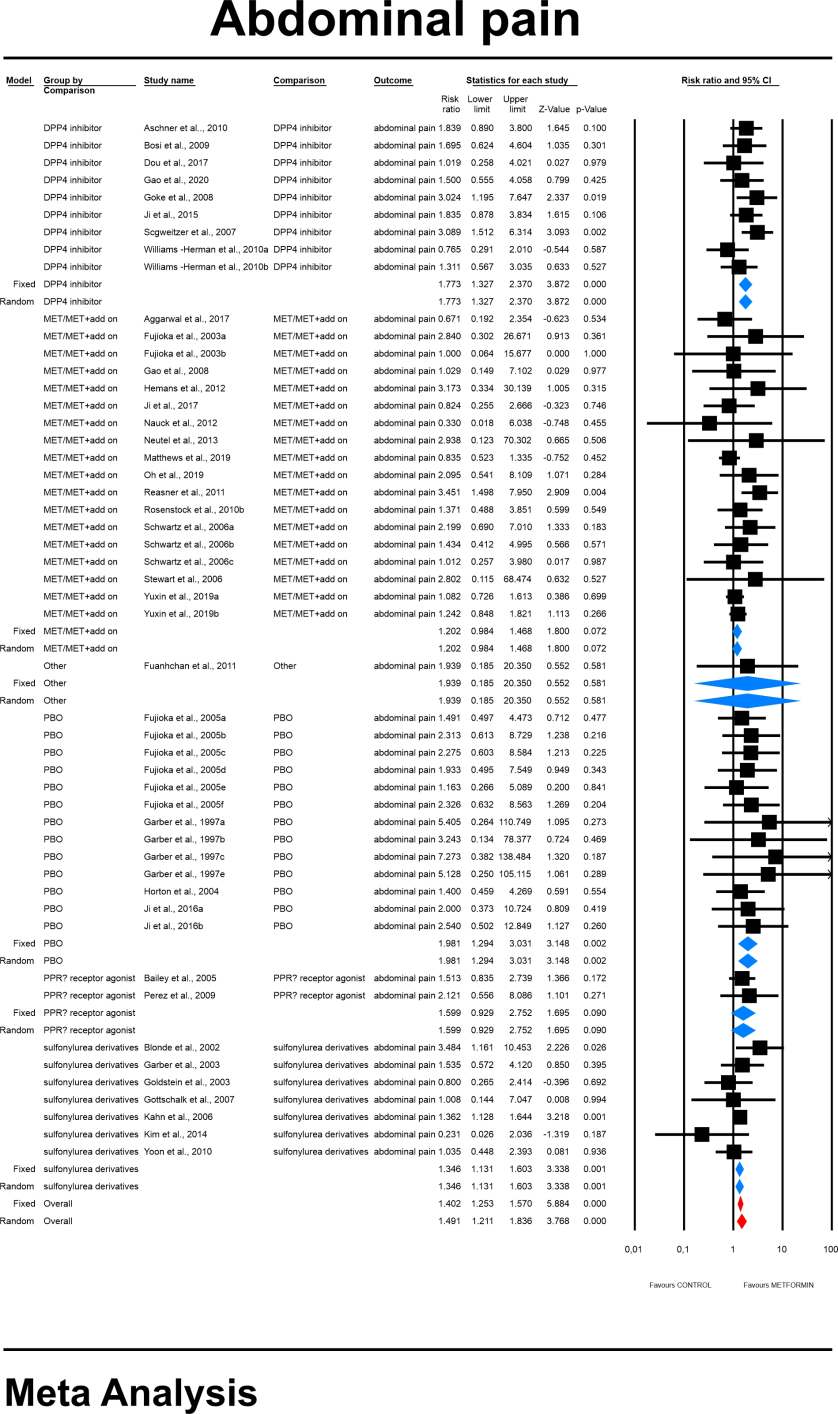
The effect size (RR) for the abdominal pain in patients taking metformin (intervention) vs. comparator (control).

Using random-effects weights, we found that the overall risk for abdominal pain was about 50% higher in patients treated with metformin compared to controls (RR=1.491, 95%CI [1.211, 1.836], p=0.0001). This was not changed when a study in children was excluded. Subgroup analysis demonstrated that the risk toward abdominal pain was significantly different regarding comparators used with the highest risk of the outcome in comparison to placebo (RR=1.981, 95%CI [1.294, 3.031], p=0.0002, **Figure 2**).

In meta-regression we found that neither the dose of metformin (coefficient =0.0002; standard error (SE) = 0.0001, Z =1.62, *p* = 0.106) (ESM **Figure S1**) nor the duration of the trial (coefficient = -0.0001; SE = 0.0001, Z=-1.67, *p* = 0.095) (ESM **Figure S2**) and type of metformin used (XR coefficient 0.059, SE = 0.195, Z=0.3, p=0.76) (ESM **Figure S3**) along with pre-existence of MET treatment: (STARTED coefficient =0.18151; SE = 0.1744, Z =1.04, *p* = 0.2980) and ethnicity of the participants (white coefficient =0.0799; SE = 0.3394, Z =0.24, *p* = 0.8138; diverse coefficient =0.1255; SE = 0.3461, Z =0.36, *p* = 0.7169) (ESM **Figure S4**) influenced study level effect sizes. Finally, we inspected funnel plots and found that Egger’s test did not suggest a publication bias regarding the RR of abdominal pain (p=0.06) (ESM **Figure S5**).

#### 4.3.2 Bloating risk

**Figure 3:**
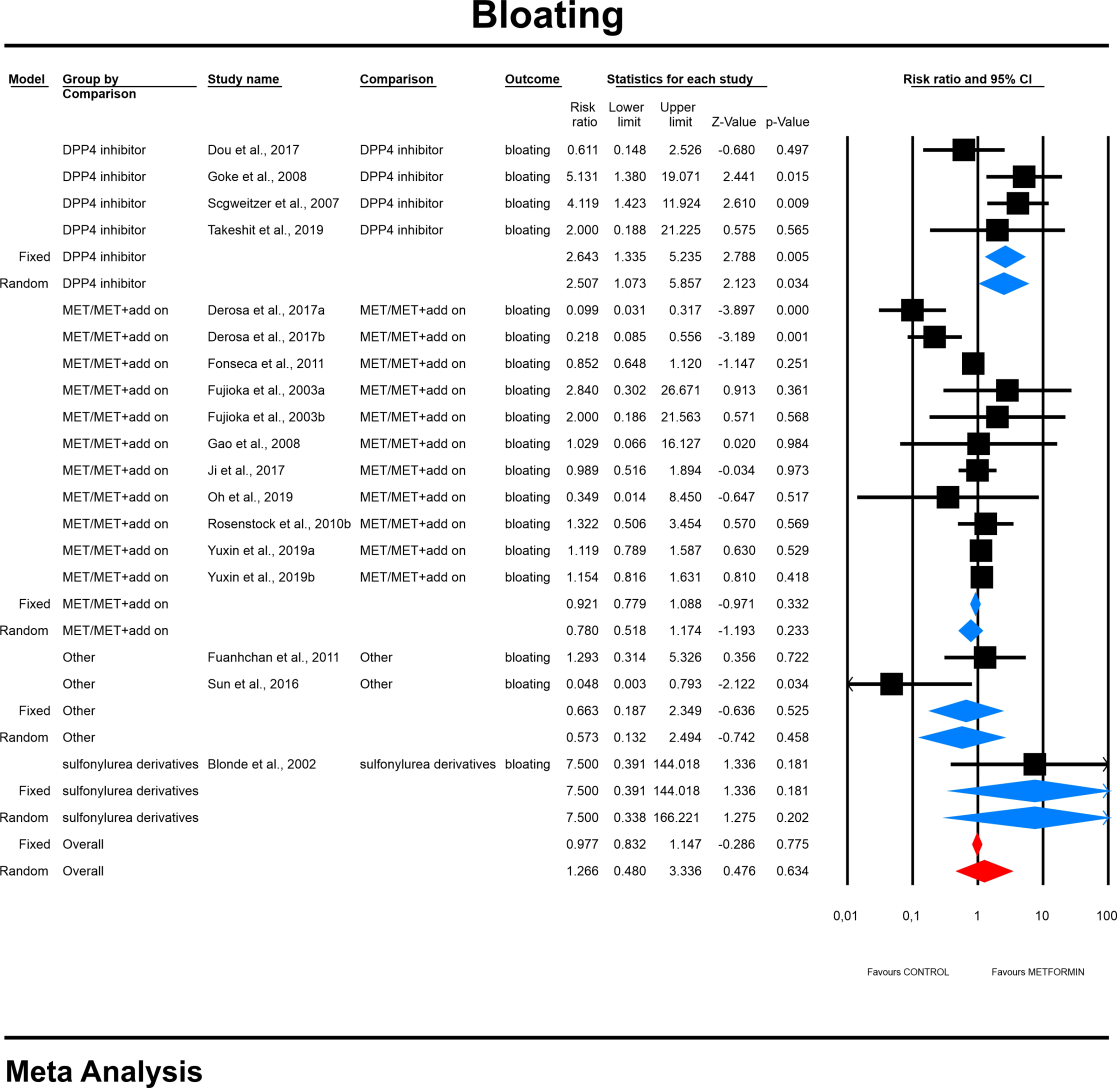
The effect size (RR) for bloating in patients taking metformin (intervention) vs. comparator (control).

**Figure 4:**
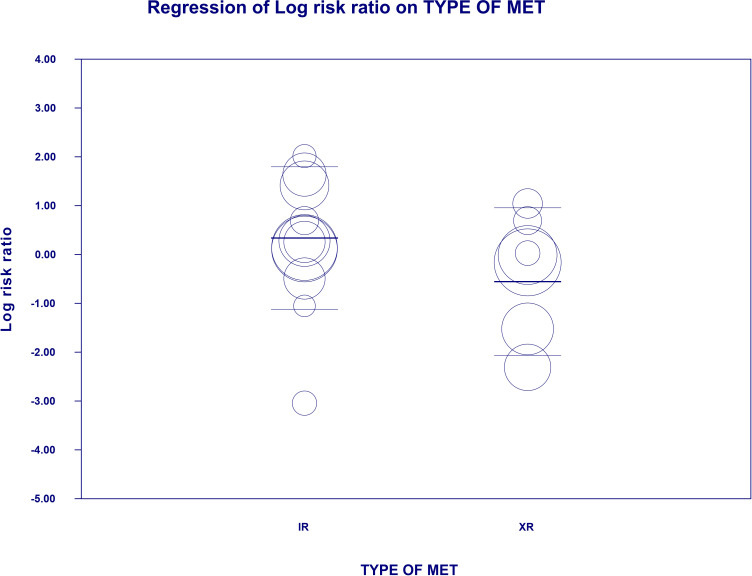
Regression for RR toward bloating by type of metformin. Each trial is represented by a circle, which size reflects the influence of that study on the model (size is inversely proportional to the variance of study). Horizontal lines are estimates of the effect size and 95% confidence intervals. Risk ratio is showed on a logarithmic scale. XR, extended release; IR, immediate release.

The overall risk for bloating was not significantly different compared to all controls (RR=1.266, 95%CI [0.48, 3.336], p=0.634), although differed significantly by each comparator type. The subgroup analyses revealed that the risk toward this outcome in MET treated patients was more than two times elevated when compared to patients receiving DPP4i (RR=2.507, 95%CI [1.073, 5.857], p=0.034; **Figure 3**). On meta-regression, four of the covariates did not influence the effect size in case of bloating; dosage: coefficient =0.0002; SE = 0.0004, Z =0.6, *p* = 0.551 (ESM **Figure S6**); duration of intervention: coefficient =0.003; SE = 0.0022, Z =1.36, *p* = 0.17 (ESM **Figure S7**); pre-existence of MET treatment: STARTED coefficient =-0.7046; SE = 0.5182, Z =-1.36, *p* = 0.1739; ethnicity of the participants: white coefficient =1.6067; SE = 1.7846, Z =0.90, *p* = 0.3680; diverse coefficient =0.4591; SE = 1.5881, Z =0.29, *p* = 0.7725) (ESM **Figure S8**). Bloating risk was higher in persons receiving IR metformin when compared to XR drug (XR: coefficient -0.89, SE = 0.195, Z=0.3, p=0.76) (**Figure 4**). Finally, we inspected funnel plots to find that Egger’s test did not suggest a publication bias regarding the RR of bloating (p=0.996) (ESM **Figure S9**).

#### 4.3.3 Constipation risk

**Figure 5:**
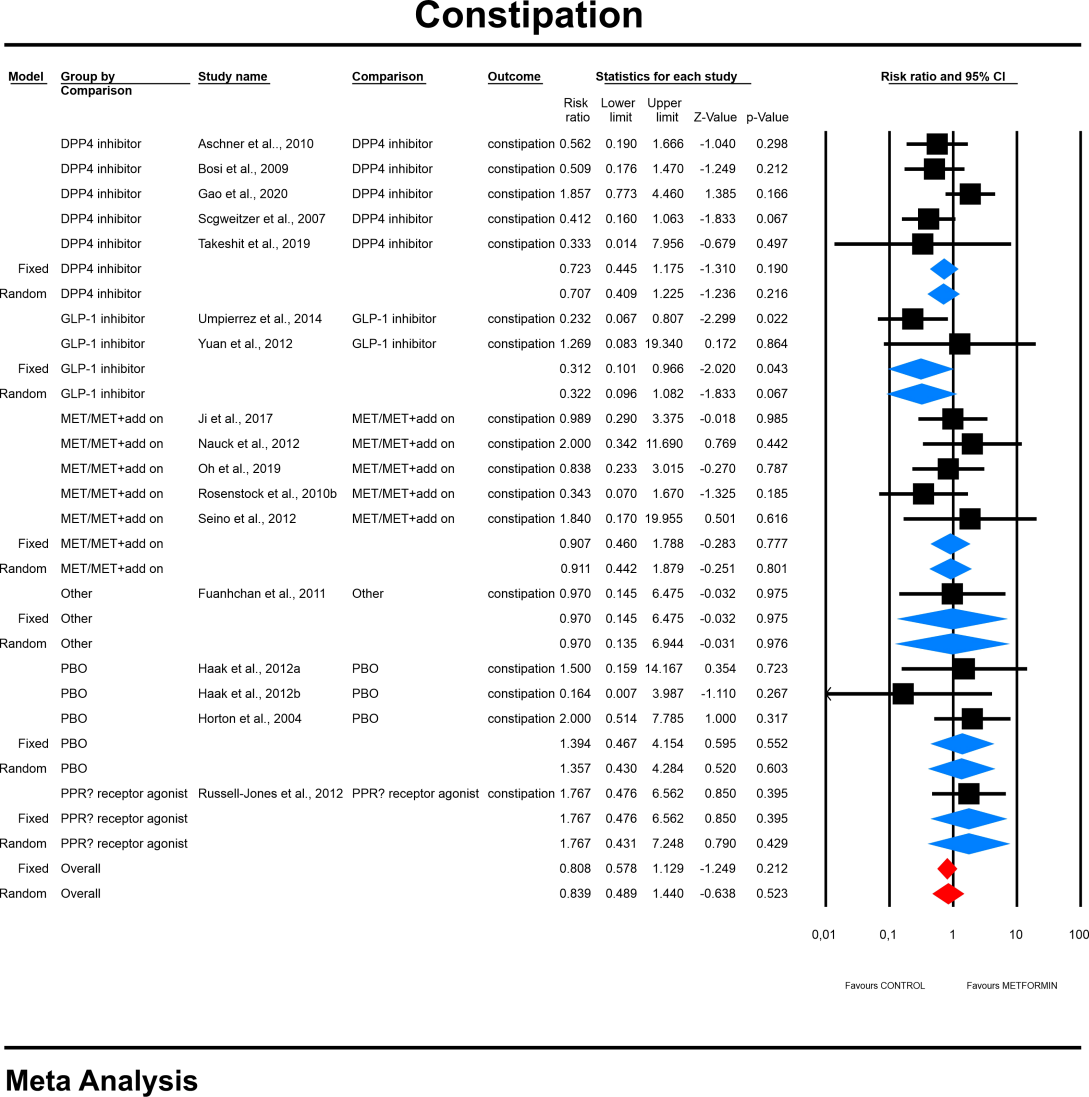
The effect size (RR) for constipation in patients taking metformin (intervention) vs. comparator (control).

**Figure 6:**
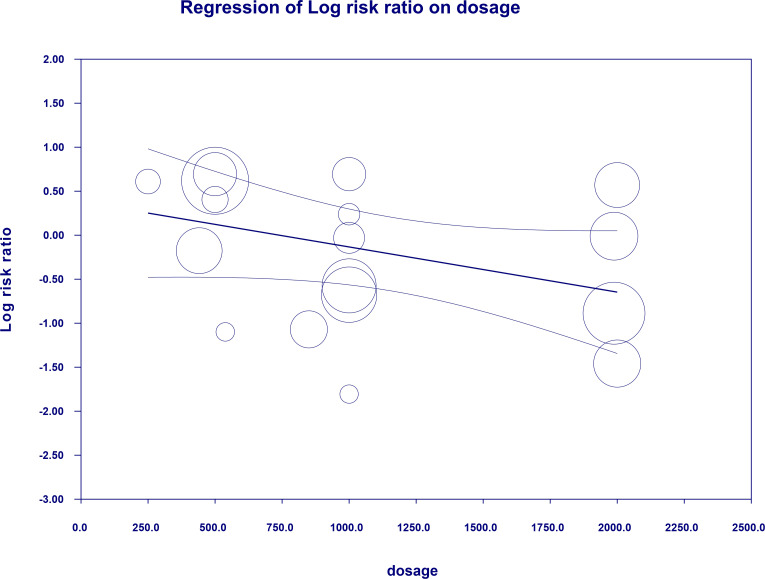
Regression for RR toward constipation by dosage.

The overall risk for constipation was not significantly different when compared to controls (RR=0.839, 95%CI [0.489, 1.440], p=0.523). The risk did not differ significantly in a subgroup analyses (**Figure 5**). In case of meta-regression we found that dosage was tended to be negatively linked to the effect size; dosage: coefficient =-0.0005; standard error (SE) = 0.0003, Z =-1.90, *p* = 0.057 (**Figure 6**) and participants of white ethnicity had higher constipation risk (white: coefficient =1.3873; SE = 0.9369, Z =1.48, *p* = 0.0370) (ESM **Figure S10**). The duration of an intervention (coefficient =-0.001; standard error (SE) = 0.0013, Z =-0.78, *p* = 0.43) (ESM **Figure S11**), type of metformin (XR: coefficient 0.219, SE = 0.727, Z=0.3, p=0.76) (ESM **Figure S12**) and pre-existence of MET treatment (STARTED coefficient =-0.9335; SE = 0.7635, Z =-1.22, *p* = 0.2215) did not influence the effect size significantly. Egger’s test did not suggest a publication bias regarding the RR of constipation (p=0.99) (ESM **Figure S13**).

#### 4.3.4 Diarrhea risk

**Figure 7:**
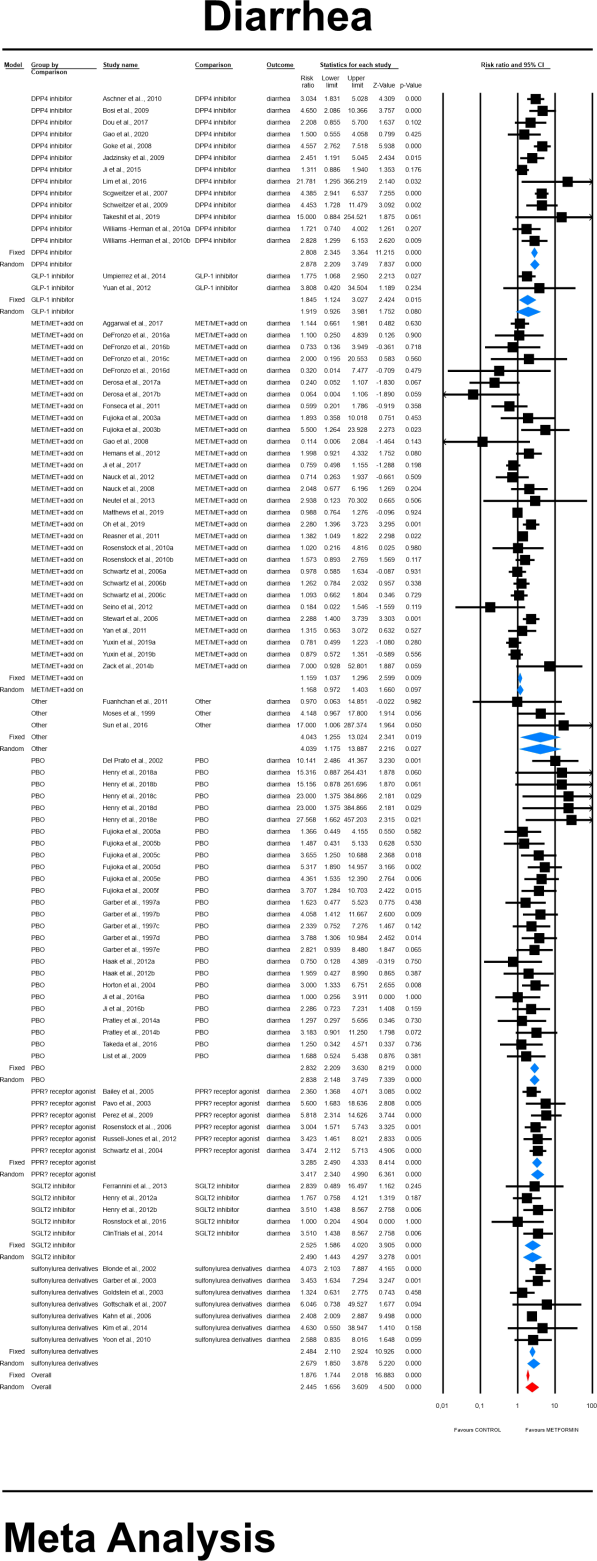
The effect size (RR) for diarrhea in patients taking metformin (intervention) vs. comparator (control).

**Figure 8:**
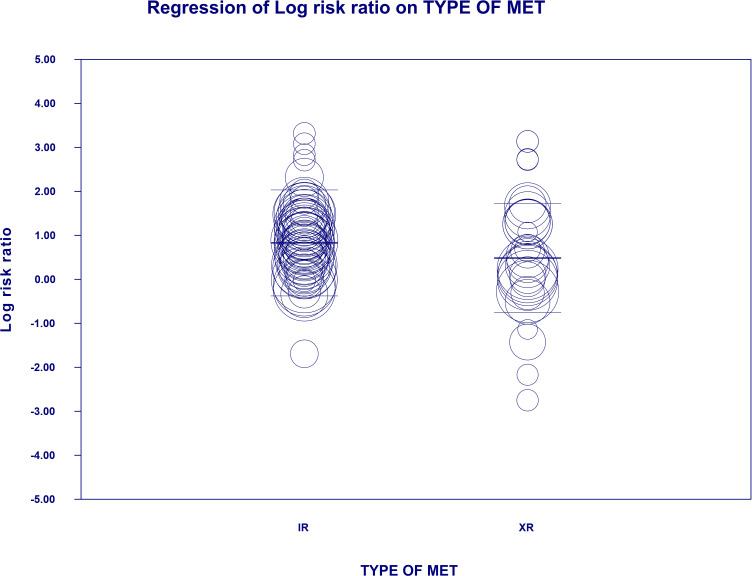
Regression for RR toward diarrhea by type of metformin. XR, extended release; IR, immediate release.

**Figure 9:**
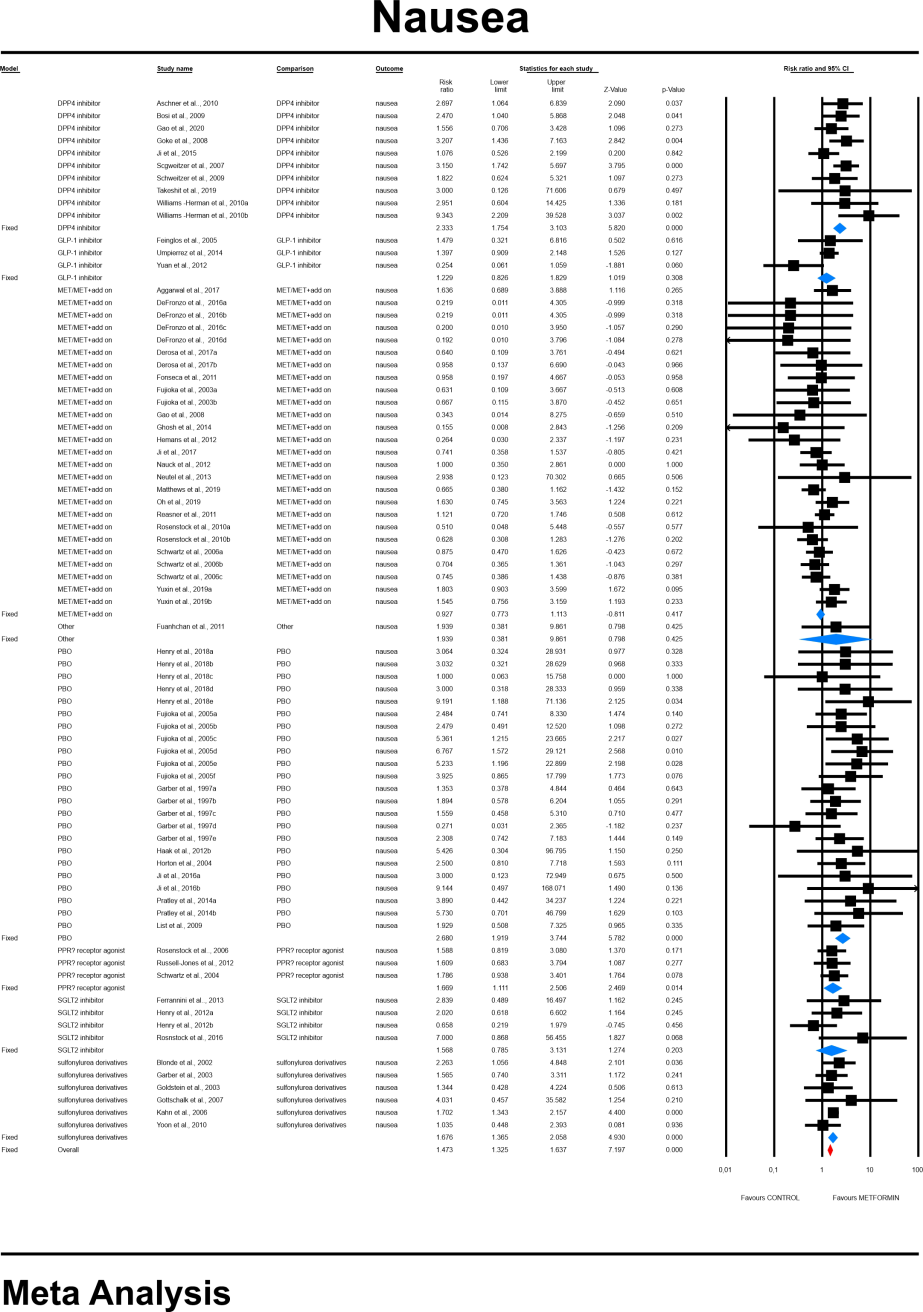
The effect size (RR) for nausea in patients taking metformin (intervention) vs. comparator (control).

**Figure 10:**
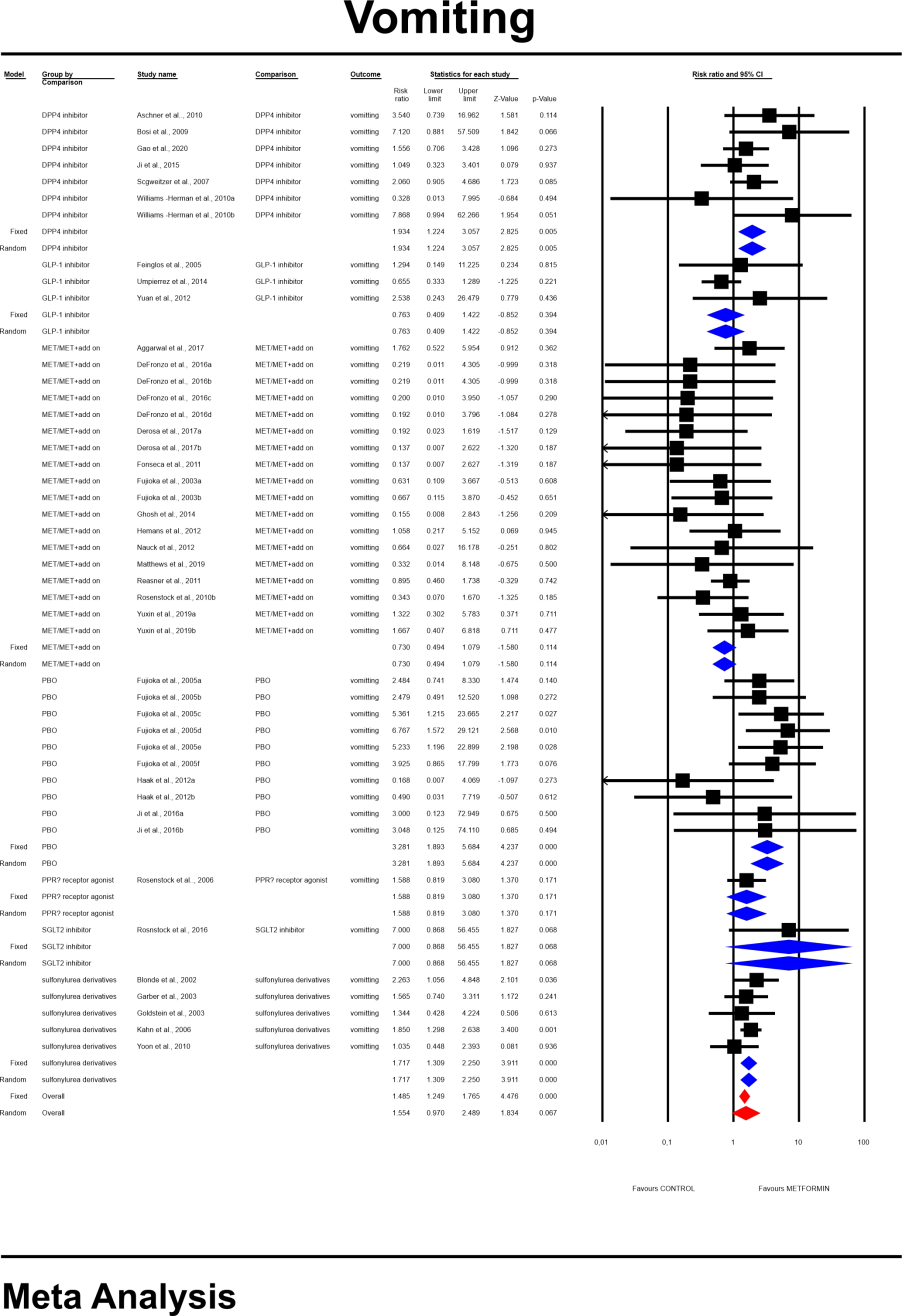
The effect size (RR) for vomiting in patients taking metformin (intervention) vs. comparator (control).

The overall risk for diarrhea was significantly elevated compared to all controls (RR=2.445, 95%CI [1.656, 3.609], p=0.0001), and differed significantly by each comparator type. This was not changed when a study in children was excluded. The subgroup analyses revealed that highest risk for diarrhea in patients receiving metformin was demonstrated in comparison to glinides and acarbose (Category: Other) (RR=4.039, 95%CI [1.175, 13.887], p=0.0027; **Figure 7**). In case of meta-regression, four of the covariates did not influence the effect size; dosage: coefficient =0.0001; SE = 0.0001, Z =0.6, *p* = 0.549 (ESM **Figure S14**); duration of intervention: coefficient =-0.0001; SE = 0.0002, Z =-0.59, *p* = 0.557 (ESM **Figure S15**); pre-existence of MET treatment: STARTED coefficient =0.29391; SE = 0.2103, Z =1.40, *p* = 0.1623; ethnicity of the participants: white coefficient =0.3261; SE = 0.4460, Z =0.73, *p* = 0.4646; diverse coefficient =0.2331; SE = 0.3960, Z =0.59, *p* = 0.5560 (ESM **Figure S16**). In contrast, we found that the risk of diarrhea was elevated in persons receiving IR metformin (XR: coefficient –0.344, SE = 0.171, Z=-2.02, p=0.0437). The results can be found in **Figure 8**. Egger’s test did not suggest publication bias regarding the RR of diarrheal (p=0.056) (ESM **Figure S17**) (**Figures 9**, **10**).

#### 4.3.5 Nausea risk

General risk for nausea was significantly elevated compared to all controls (RR=1.641, 95%CI [1.169, 2.302], p=0.0004), and differed significantly by each comparator type. This was not changed when a study in children was excluded. The subgroup analyses revealed that highest risk was in comparison to placebo (RR=2.608, 95%CI [1.919, 3.774], p=0.0001; **Figure 9**). In case of meta-regression, none of tested covariates influenced the effect size; dosage: coefficient =-0.0000; SE = 0.0001, Z =-0.06, *p* = 0.953 (ESM **Figure S18**); duration of intervention: coefficient =-0.0001; SE = 0.0002, Z =-0.44, *p* = 0.659 (ESM **Figure S19**); type of metformin: XR: coefficient -0.296, SE = 0.168, Z=-1.76, p=0.07 (ESM **Figure S20**); ethnicity of the participants: white coefficient =0.2654; SE = 0.5484, Z =0.48, *p* = 0.6284; diverse coefficient =0.1353; SE = 0.4900, Z =0.28, *p* = 0.7824 (ESM **Figure S21**). In persons that started the MET treatment in a trial we found a statistical tendency to manifest nausea more frequently compared to patients in whom MET was given before (STARTED: coefficient=0.4347; SE=0.2422, Z=1.79, p=0.0727). Egger’s test did not suggest publication bias regarding the RR of nausea (p=0.613) (ESM **Figure S22**).

#### 4.3.6 Vomiting risk

The overall risk for vomiting was not significantly different when compared to controls (RR=1.554, 95%CI [0.970, 2.489], p=0.067). The risk however differ significantly in a subgroup analyses (**Figure 10**). None of tested covariates significantly linked to the effect size; dosage: coefficient =0.0002; standard error (SE) = 0.0002, Z =0.96, *p* = 0.339 (ESM **Figure S23**); the duration of an intervention (coefficient =0.001; standard error (SE) = 0.0003, Z =0.31, *p* = 0.76) (ESM **Figure S24**), type of metformin (XR: coefficient 0.107, SE = 0.282, Z=0.38, p=0.704) (ESM **Figure S25**). and ethnicity of the participants (white: coefficient =0.1958; SE = 0.6709, Z =0.29, *p* = 0.7704) (ESM **Figure S26**). However, in persons that started the MET treatment in a trial we found a statistical tendency to manifest this GI event more frequently compared to patients in whom MET was given before (STARTED: coefficient=0.803; SE=0.4498, Z=1.79, p=0.0742). Egger’s test did not suggest a publication bias regarding the RR of nausea (p=0.11) (ESM **Figure S27**).

The risk of discontinuing the study due to adverse events

For all studies included, the risk for discontinuing the study was comparable between groups and did not differ significantly; RR: 1.080, 95%CI [0.949, 1.228], p=0.243.

### 4.4 The Risk of bias of included studies

By means of Cochrane’s collaboration tool, we estimated that the mean number of low risk of bias assessments was 4.42 ± 1.42 (median 5). The highest score, i.e. 7 low ROB assessments were demonstrated for 5 studies (27–31) whilst only one study presented lowest score (32). The details are presented in ESM **Table S5**.

## 5 Discussion

In this systematic review and meta-analysis with meta-regression our principal findings are as follows: (i) the risk of abdominal pain, nausea and diarrhea was higher in T2DM patients treated with metformin compared to other antidiabetic drugs or placebo; (ii) there is no significant risk of GI AEs associated neither with the dose size of metformin nor metformin treatment duration; and (iii) metformin XR formulation is associated with lower risk of bloating and diarrhea compared to metformin IR.

While interpreting these data, it is important to note, that a low number of studies comparing metformin and SGLT-2i or GLP-1RA were included, especially reporting GI AE other than diarrhea. A heterogeneous group of studies comparing metformin formulations or metformin with a combination of metformin and other antidiabetic drugs (MET/MET+add on) showed no differences in GI AE risk. This result suggests that the addition of another antidiabetic drug to metformin does not increase GI AE risk compared to metformin monotherapy, however, arms with an additional antidiabetic drug often provided lower doses of metformin than arms using metformin alone. The highest risk of abdominal pain and nausea was present when metformin was compared to placebo and the highest risk of diarrhea was seen when metformin was compared to glinides and acarbose. The latter outcome implies that diarrhea might be one of the strongest GI AE of metformin administration since it exerts the highest risk even when compared to the alpha-glucosidase inhibitor for which diarrhea is well recognized AE (33). The reasons why metformin leads to diarrhea might be related to some structural similarities with agonists of the 5-HT3 receptor (serotonergic-like effect of metformin) since serotonin (5-HT) released from the intestine may cause GI symptoms like diarrhea, nausea and vomiting (10, 34) There is also a hypothesis that genetic variations in OCT1 are likely to be involved in the absorption of metformin from the intestinal lumen, whereby reduced transport by this transporter might increase metformin concentrations in the intestine in prone individuals leading to increased GI AE (13, 14). Moreover, metformin leads to reduction of ileal absorption of bile acids what may cause osmotic diarrhea (12).

In previous network meta-analyses based on studies comparing DPP-4i or GLP-1RA with other hypoglycemic drugs, treatment with metformin, GLP-1RA and acarbose were ranked as having the highest incidence of GI AEs, while that of glitazones, sulfonylurea derivatives, SGLT-2i and DPP-4i was comparable or lower than placebo (21, 22). To assess the severity of GI AEs and their possible effect on treatment compliance, withdrawal rates were analyzed, but no significant differences between arms were found.

To facilitate evidence-informed decision-making regarding metformin treatment, associations between metformin dose size, type of formulation used (IR or XR), ethnicity of participants and GI AE risk were investigated with meta-regression.

### 5.1 Metformin dose size

Advice from the American Diabetes Association (ADA) and the European Association for the Study of Diabetes (EASD) (35) is to lower the metformin dose, when the GI adverse symptoms occur, with the belief that the symptoms will resolute with time. Evidence regarding dose-dependency of GI AEs is limited. Although in few studies there is a numerical increase in GI AEs with increasing dose of metformin, this finding was either not consistent across all doses used in a study (36), reported as non-significant (37, 38) or authors did not comment on its significance (39). Most notably, trials designed to study the safety of different dosages of metformin did not find the relationship between dosage and incidence of GI AEs (40, 41). Other studies had similar conclusions (42, 43). In relation to dosage of metformin in our analysis association trended towards significance (p=0.057) only with constipation (the higher the dose the lower its risk).

### 5.2 Metformin treatment duration and prior metformin treatment

Whether the duration of metformin treatment has an impact on the incidence of GI AEs is unknown, however, GI AEs may improve over time (35). Yuxin et al. (41) reported that the vast majority of discontinuations of treatment due to metformin intolerance occurred in the first third of the length of the trial (41). These data suggest no correlation between withdrawal rate due to AEs and treatment duration, as most occur only in the initial phase of therapy. In our analysis, the duration of treatment did not influence the risk of any GI AE. Initiation of metformin treatment in a trial was not associated with an increased risk of any GI AE compared to continuation of existing metformin therapy. However, there was a statistical tendency for increased risk in case of nausea and vomiting.

### 5.3 Formulation of metformin

There are two metformin formulations widely used in clinical practice, namely metformin IR and metformin XR however the rationale for choosing one formulation over the other has not been definitely proved. International guidelines for diabetes treatment like EASD/ADA (35) indicate that there is no difference in side effect profile between the two formulations of metformin, based on one randomized, controlled study (44), not metanalysis of randomized controlled trials.

The UK NICE guidelines recommend the use of metformin XR in patients intolerant to metformin IR (45). Another formulation of metformin – delayed-release (DR) was also developed to maximize gut-based mechanism of action and decrease plasma concentration, thus increasing efficacy and possibly ameliorating AEs (46). We show that the type of formulation used had a significant meaning in case of bloating and diarrhea where treatment with metformin IR was associated with a higher risk of certain GI AEs. The latter finding is reflected in the previous metanalysis showing that metformin XR compared to metformin IR is associated with a reduction of GI adverse side effects, but did not reach the pre-specified threshold for statistical significance (18). On the other hand, there was similar effectiveness and safety of metformin XR and IR, suggesting that it might not be appropriate to switch from metformin IR to metformin XR for improving glucose control or reducing AEs (19). In the present meta-analysis, we analyzed the impact of metformin formulation on effect estimates by means of meta-regression. This approach takes into account more studies that used metformin XR, but without direct comparison with metformin IR.

### 5.4 Ethnicity

In this meta-analysis, the ethnicity of trial participants was not associated with an increased risk of GI AE, except for higher risk of constipation in white individuals. This result, however, stems only from one trial classified as having participants of white ethnicity.

### 5.5 Strengths and limitations

To the best of our knowledge this analysis provides the first systematic review with meta-analysis and meta-regression of RCT regarding the risk of GI AEs in patients with T2DM treated with metformin. The limitations of the performed analysis are that some factors that could influence the tolerability of drugs in certain studies such as concomitant treatment (other than anti-diabetic) were not included in meta-regression. This could be especially important for drugs inhibiting organic cation transporter 1 (e.g. proton pump inhibitors, tricyclic antidepressants, clopidogrel) which use has been shown to be associated with metformin intolerance (13). Many excluded studies did not report detailed statistics regarding GI AEs or omitted to report any AEs what limited amount of data which could be analyzed. Some of the included studies presented data on only few GI AEs, hence there is missing data related to components of our primary outcome. Studies almost universally did not specify definitions of GI AEs and method of assessment (presumably patient reported). Differences in that aspect could explain substantial differences in absolute risk of GI AEs occurrence between studies, however, this meta-analysis regarded only relative risks and heterogeneity among studies was not high. Meta-regression was utilized to find answers to clinically relevant questions regarding metformin intolerance. Nevertheless, associations between average patient characteristics and pooled occurrence of AEs may not reflect true associations between patient-level characteristics and outcome.

## 6 Conclusion

The risk of GI AEs such as abdominal pain, nausea and diarrhea is higher in T2DM patients treated with metformin compared to other antidiabetic drugs or placebo. There is a higher risk of bloating and diarrhea with metformin IR than with metformin XR formulation. Neither the increasing dose size of metformin nor longer metformin treatment duration increases the risk of GI AEs.

## Data availability statement

The original contributions presented in the study are included in the article/**Supplementary Material**. Further inquiries can be directed to the corresponding author.

## Author contributions

KN and KS-Ż were responsible for the concept and design of the study. KN, KS-Ż, IŁ, JG, and GL supervised the study. MH, KI, and KN contributed to study selection, eligibility check and data extraction. HK, KJ, and IŁ assessed the risk of bias. KN was a clinical leader. KS-Ż carried out statistical analysis. The manuscript was drafted by KN, KS-Ż, and KI. All authors revised the manuscript and approved the final version.

## Funding

This research received no specific grant from any funding agency in the public, commercial or not-for-profit sectors.

## Conflict of interest

The authors declare that the research was conducted in the absence of any commercial or financial relationships that could be construed as a potential conflict of interest.

## Publisher’s note

All claims expressed in this article are solely those of the authors and do not necessarily represent those of their affiliated organizations, or those of the publisher, the editors and the reviewers. Any product that may be evaluated in this article, or claim that may be made by its manufacturer, is not guaranteed or endorsed by the publisher.
