# Supplementary material for: Gastrointestinal adverse events of metformin treatment in patients with type 2 diabetes mellitus: A systematic review, meta-analysis and meta-regression of randomized controlled trials
Source: Front Endocrinol (Lausanne). 2022 Sep 14;13:975912. doi: 10.3389/fendo.2022.975912 (PMC9524196; doi:10.3389/fendo.2022.975912)
Supplement: Supplementary file 1 [file DataSheet_1.docx]

Supplementary Material

# Supplementary Data

**ELECTRONIC SUPPLEMENTARY MATERIAL:**

**Search Strategy and Inclusion/Exclusion Criteria**

There were two independent authors (KI and MH) who searched five certain bibliographic databases (PUB MED/Cinahl/Web of Science/Scopus/Embase) from database inception until 08/11/2020 for studies evaluating GI AEs of metformin treatment in patients with type 2 diabetes. Our target was data provided in randomized controlled trials only.

The following search terms were used:

PUB MED/Cinahl/Web of Science/Scopus/

(diabetes mellitus OR diabetes OR diabetes mellitus OR diabetic OR insulin resistance OR insulin resistance OR resistance, insuline) AND (metformin OR 1, 1 dimethylbiguanide OR dimethyldiguanide OR dimethylbiguanide OR metformin OR metformina OR metformine OR methformin OR metiguanide OR metphormin OR n` dimethylguanylguanide OR n` dimethylguanylguanidine OR n`, n` dimethyldiguanide OR n, n dimethyl biguanidine OR n, n dimethylbiguanide OR n, n dimethyldiguanide OR n, n dimethylbiguanidine OR n, n dimethylbiguanide retard OR n, n dimethylguanylguanidine) AND (abdominal pain OR abdomen pain OR abdominal pain OR pain, abdominal OR nausea and vomiting OR nausea and emesis OR nausea and vomiting OR nausea emesis OR bloating OR abdominal bloating OR bloating OR diarrhea OR diarrhea OR diarrhea, toxic OR diarrhoea OR diarrhoea, toxic OR postoperative diarrhea OR postoperative diarrhoea OR scour OR toxic diarrhea OR toxic diarrhoea) NOT (review OR meta analysis OR systematic review)

EMBASE

('diabetes mellitus' OR 'diabetes' OR 'diabetes mellitus' OR 'diabetic' OR 'insulin resistance' OR 'insulin resistance' OR 'resistance, insuline') AND ('metformin' OR '1, 1 dimethylbiguanide' OR 'dimethyldiguanide' OR 'dimethylbiguanide' OR 'metformin' OR 'metformina' OR 'metformine' OR 'methformin' OR 'metiguanide' OR 'metphormin' OR 'n` dimethylguanylguanide' OR 'n` dimethylguanylguanidine' OR 'n`, n` dimethyldiguanide' OR 'n, n dimethyl biguanidine' OR 'n, n dimethylbiguanide' OR 'n, n dimethyldiguanide' OR 'n, n dimethylbiguanidine' OR 'n, n dimethylbiguanide retard' OR 'n, n dimethylguanylguanidine') AND ('abdominal pain' OR 'abdomen pain' OR 'abdominal pain' OR 'pain, abdominal' OR 'nausea and vomiting' OR 'nausea and emesis' OR 'nausea and vomiting' OR 'nauseaemesis' OR 'bloating' OR 'abdominal bloating' OR 'bloating' OR 'diarrhea' OR 'diarrhea' OR 'diarrhea, toxic' OR 'diarrhoea' OR 'diarrhoea, toxic' OR 'postoperative diarrhea' OR 'postoperative diarrhoea' OR 'scour' OR 'toxic diarrhea' OR 'toxic diarrhoea') NOT ('review' OR 'meta analysis' OR 'systematic review')

ClinTrials: metformin | Completed Studies | Diabetes Mellitus (completed studies as a filter)

**Inclusion criteria:**

- randomized controlled trial with MET intervention in patients with type 2 diabetes mellitus
- populations containing >20 patients,
- abstraction of data from metformin-treatment arms only (MET-treatment), however in the case of multiple arms data from all containing MET-treatment were abstracted
- available data on the incidence of any gastrointestinal adverse event following metformin administration, such as abdominal pain, diarrhea, bloating, flatulence, constipation, nausea and vomiting

**Exclusion criteria:**

- adverse events related to metformin treatment other than gastrointestinal
- another than English language of publication,

**Decisions made during data extraction that did not result from inclusion or exclusion criteria.**

If a trial published multiple reports with different follow up duration, data in the report with the longest follow-up, without treatment regimen change in metformin arm or comparator arm according to protocol, was extracted. If a trial had more than one comparator arm, we extracted data from only one of the arms, choosing placebo if available, sulfonylureas instead of thiazolidinediones and thiazolidinediones instead of dipeptidyl peptidase-4 inhibitors (DPP4i). Whenever more than one dose of active comparator was investigated, we collected data only from the arm with the lowest dose used. In case multiple doses of metformin were used during a trial we utilized the mean dose achieved in the arm. The trial population was considered to be White or Asian if at least 88% of the population was described as such, otherwise, it was labelled as diverse.

ESM Figure S1

ESM Figure S2

ESM Figure S3

ESM Figure S4 ESM Figure S5

ESM Figure S6

ESM Figure S7

ESM Figure S8 ESM Figure S9

ESM Figure S10

ESM Figure S11

ESM Figure S12 ESM Figure S13

ESM Figure S14

ESM Figure S15

ESM Figure S16 ESM Figure S17

ESM Figure S18

ESM Figure S19

ESM Figure S20

ESM Figure S21 ESM Figure S22

ESM Figure S23

ESM Figure S24

ESM Figure S25

ESM Figure S26 ESM Figure S27

ESM Table S1. Studies included comparing metformin with placebo or active comparator

| **Study characteristics** | | | | **MET treatment** | | | | | | **comparator** | | **Patients characteristics** | | | **BMI (kg/m2) at baseline** | |  |
| --- | --- | --- | --- | --- | --- | --- | --- | --- | --- | --- | --- | --- | --- | --- | --- | --- | --- |
| **Reference** | **ITT/PP** | **Focus of the study** | **N total randomized/**  **analyzed** | **MET (P/S)/p dose** | **MET commercial labeling** | **Dosage (unit/day)** | **Duration (days)** | **MET XR/IR** | **Active arm** |  | **age (Ø ±SD)** | | **Male (N/%)** | **MetforminØ±SD** | | **Control Ø±SD** | |
| Aschner et al., 2010 [1] | ITT | To compare the efficacy and safety of monotherapy with sitagliptin and metformin in treatment‐naïve patients with T2DM. | 1050/1050 | S | nd | 2000mg/2/d | 168 | IR | Metformin IR | sitagliptin/DPP4 inhibitor | 56±10.5 | | 411/46 | 30.9±4.9 | | 30.7±4.7 | |
| Blonde et al., 2002 [2] | ITT | To compare the efficacy, safety and tolerability of glyburide alone or metformin alone in patients with T2DM inadequately controlled by sulphonylurea, diet and exercise. | 639/639 | S | nd | 1840mg/nd | 112 | IR | Metformin IR | glyburide/sulfonylurea derivatives | 56.7±9.2 | | 189/59.6 | 30.6±4.4 | | 30.3±4.4 | |
| Ferrannini et al.., 2013 [3] | ITT | To investigate the long-term safety and efficacy of empagliflozin, a sodium glucose cotransporter 2 inhibitor and metformin monotherapies in patients with T2DM. | 659/659 | S (study1)/ P (study2)≥10 weeks≥1,5 g/d | nd | 2000mg/2/d | 546 | IR | Metformin IR | Empagliflozin 10 mg/SGLT2 inhibitor | 57.78±8.67 | | 77/47.5 | 28.6± 22.4-39.3 | | 28.9± 20.3-39.2 | |
| Bosi et al., 2009 [4] | ITT | To compare the efficacy and safety of vildagliptin and metformin individual monotherapies in treatment‐naive patients with T2DM | 1179/1171 | S | nd | 2000mg/2/d | 168 | IR | Metformin IR | Vildagliptin/DPP4 inhibitor | 53±10.9 | | 351/59.1 | 31.31±4.58 | | 31.26±4.82 | |
| DeFronzo et al., 1995 [5] | ITT | To test the efficacy of Metformin in Patients with Non-Insulin-Dependent Diabetes Mellitus | 289/289 | S | nd | 2550mg/3/d | 203 | IR | Metformin IR | PBO | 53±12.02 | | 124/42.9 | 29.9±3.6 | | 29.2±3.6 | |
| Del Prato et al., 2002 [6] | ITT | To compare the efficacy of six-month therapy of benfluorex (Mediator) (150–450 mg/day) vs. placebo or metformin hydrochloride | 722/722 | S | nd | 2125mg/2,5/d | 203 | IR | Metformin IR | PBO | 56±9 | | 259/60.5 | 29.7±4.2 | | 29.9±3.9 | |
| Dou et al., 2017 [7] | ITT | To assess the efficacy and safety of saxagliptin or metformin monotherapies over 24 weeks in pharmacotherapy‐naïve Chinese patients with T2DM and inadequate glycaemic control | 639/639 | S | nd | 2000mg/2/d | 168 | IR | Metformin IR | Saxagliptin/DPP4 inhibitor | 49.8±11 | | 283/67.4 | 26.5±3.6 | | 26.5±3.2 | |
| Henry et al., 2018a [8] | ITT | To evaluate the glycemic effects and safety of DR metformin delayed-release | 571/571 | S | nd | 600mg/1/d | 112 | DR | Metformin DR | PBO | 56.5±10.5 | | 91/47.9 | 33±5 | | 31±5 | |
| Henry et al., 2018b |  |  |  |  | nd | 900mg/1/d |  |  | Metformin DR |  | 56±10.6 | | 100/52.5 | 32±6 | |  |  |
| Henry et al., 2018c |  |  |  |  | nd | 1200mg/1/d |  |  | Metformin DR |  | 56±11 | | 94/49 | 32±6 | |  |  |
| Henry et al., 2018d |  |  |  |  | nd | 1500mg/1/d |  |  | Metformin DR |  | 56±10.1 | | 97/50.5 | 33±6 | |  |  |
| Henry et al., 2018e |  |  |  |  | Metformin IR Glucophage1 (Bristol-Myers Squibb; Princeton, NJ) | 2000mg/2/d |  | IR | Metformin IR |  | 57±11 | | 92/48.5 | 32±5 | |  |  |
| Feinglos et al., 2005 [9] | ITT | To explore the dose–response relationship of metformin effects on bodyweight and glycaemic control in subjects with T2DM | 210/210 | P/4 weeks/1.5g/d | Glucophage® | 2000mg/2/d | 84 | IR | Metformin IR | Liraglutide 0,045 mg/GLP-1 inhibitor | 53.3±9.2 | | 32/45.1 | 33.9±3.7 | | 34.3±4.1 | |
| Fuangchan et al., 2011 [10] | ITT | This study was conducted to assess the efficacy and safety of three doses of bitter melon compared with metformin. | 129/127 | S | nd | 1000mg/nd | 28 | IR | Metformin IR | bitter melon 500 mg/Other | 52.4±8.8 | | 17/25.8 | 24.4±3.1 | | 25±3.4 | |
| Fujioka et al., 2005a [11] | ITT | The efficacy, dose–response relationships and safety of an extended‐release formulation of metformin (Glucophage® XR) were evaluated in two double‐blind, randomized, placebo‐controlled studies of 24 and 16 weeks' duration, in patients with inadequate glycaemic control despite diet and exercise. Protocol 1 provided an evaluation of metformin XR at a commonly used dosage. | 240/238 | S | Glucophage XR | 1000mg/1/d | 168 | XR | Metformin XR | PBO | 56±11.1 | | 143/59.6 | 28.7±3.9 | | 28.9±3.5 | |
| Fujioka et al., 2005b |  |  | 742/738 |  |  | 500mg/1/d | 112 |  | Metformin XR |  | 54.5±10.5 | | 114/46.5 | 30.1±4 | | 30.7±4.1 | |
| Fujioka et al., 2005c |  |  | 742/738 |  |  | 1000mg/1/d |  |  | Metformin XR |  | 55±10 | | 127/53.6 | 30.6±4.2 | | 30.7±4.1 | |
| Fujioka et al., 2005d |  |  | 742/738 |  |  | 1500mg/1/d |  |  | Metformin XR |  | 55±10.6 | | 115/48.5 | 29.7±4 | | 30.7±4.1 | |
| Fujioka et al., 2005e |  |  | 742/738 |  |  | 2000mg/1/d |  |  | Metformin XR |  | 54.5±10.6 | | 112/44.6 | 30.9±4 | | 30.7±4.1 | |
| Fujioka et al., 2005f |  |  | 742/738 |  |  | 2000mg/2/d |  |  | Metformin XR |  | 55.5±10.1 | | 115/47.9 | 30.6±4.6 | | 30.7±4.1 | |
| Garber et al., 1997a [12] | ITT | To study the efficacy and safety of various dosages of metformin in patients with T2DM | 451/451 | S | nd | 500mg/1/d | 77 | IR | Metformin IR | PBO | 56±10.6 | | 89/58.6 | 31.9±nd | | 32.21±nd | |
| Garber et al., 1997b |  |  |  |  |  | 1000mg/2/d |  |  | Metformin IR |  | 55±10.5 | | 84/55.3 | 31.9±nd | |  |  |
| Garber et al., 1997c |  |  |  |  |  | 1500mg/3/d |  |  | Metformin IR |  | 57±10.7 | | 92/59.4 | 31.7±nd | |  |  |
| Garber et al., 1997d |  |  |  |  |  | 1000mg/2/d |  |  | Metformin IR |  | 57.4±11.3 | | 83/54.6 | 31.04±nd | |  |  |
| Garber et al., 1997e |  |  |  |  |  | 2500mg/3/d |  |  | Metformin IR |  | 57±11.2 | | 94/60.3 | 33.48±nd | |  |  |
| Garber et al., 2003 [13] | ITT | To compare the benefits of initial therapy with glyburide/metformin tablets vs. metformin or glyburide monotherapy | 486/485 | S | nd | 1796mg/nd | 112 | IR | Metformin IR | glyburide/sulfonylurea derivatives | 55±12 | | 137/43.5 | 31.4±4 | | 31.1±4.3 | |
| Göke et al., 2008 [14] | ITT | To compare the effi cacy and tolerability of vildagliptin and metformin in drug-naive patients with T2DM over 104 weeks. | 463/462 | P/≥52/2g/d | nd | 2000mg/2/d | 364 | IR | Metformin IR | vildagliptin/DPP4 inhibitor | 54±11 | | nd/nd | 32.7±5.7 | | 32.7±5.7 | |
| Goldstein et al., 2003 [15] | ITT | To asses therapy with glipizide or metformin monotherapies in patients with type 2 DM that is uncontrolled by at least half the maximum labelled daily dose of a sulfonylurea. | 247/246 | S | nd | 1928mg/2/d | 126 | IR | Metformin IR | glipizide/sulfonylurea derivatives | 57±9.4 | | 101/63.1 | 31.6±4.3 | | 30.6±4.8 | |
| Haak et al., 2012a [16] | ITT | To evaluate the efficacy and safety of metformin monotherapy in patients with T2DM. | 791/791 | S | nd | 1000mg/2/d | 168 | IR | Metformin IR | PBO | 53.8±10.7 | | 118/54.6 | 28.9±4.8 | | 28.6±5.2 | |
| Haak et al., 2012b |  |  | 791/791 | S | nd | 2000mg/2/d | 168 | IR | Metformin IR | PBO | 55.4±10.7 | | 114/52.1 | 29.5±5.3 | | 28.6/5.2 | |
| Henry et al., 2012a [17] | ITT | To compare dapagliflozin alone and metformin alone.  . | 603/598 | S | nd | 1843.6mg/2/d | 168 | XR | Metformin XR | dapagliflozin/SGLT2 inhibitor | 52.1±10 | | 187/46.3 | nd±nd | | nd±nd | |
| Henry et al., 2012b |  |  | 641/638 |  |  | 1949.7mg/2d |  |  | Metformin XR |  | 51.9±11 | | 202/47.3 |  |  |  |  |
| Horton et al., 2004 [18] | ITT | To assess the efficacy and tolerability of \ metformin (500 mg, tid) as initial treatment in drug-naive patients with T2DM (T2DM). | 701/701 | S | nd | 1500mg/3/d | 168 | IR | Metformin IR | PBO | 57.2±2.1 | | 137/65.87 | 29.9±0.4 | | 29.5±0.4 | |
| Jadzinsky et al., 2009 [19] | ITT | To evaluate the efficacy and safety of initial saxagliptin or metformin monotherapy in treatment‐naïve patients with T2DM (T2D) and inadequate glycaemic control. | 1306/1306 | S | nd | 1818mg/2/d | 168 | IR | Metformin IR | Saxagliptin/DPP4 inhibitor | 52±10.5 | | 332/50.1 | 30.2±4.9 | | 30.2±4.9 | |
| Ji et al., 2016a [20] | ITT | To evaluate the efficacy and safety of initial metformin monotherapy in Chinese patients with T2DM and inadequate glycemic control | 744/743 | S | nd | 1000mg/2/d | 168 | IR | Metformin IR | PBO | 53.1±9.6 | | 156/61.7 | 26±3.7 | | 25.4±3.4 | |
| Ji et al., 2016b |  |  |  |  |  | 1700mg/2/d |  |  | Metformin IR |  | 53.3±10 | | 162/64.5 |  | | 25.4±3.4 | |
| Kahn et al., 2006 [21] | ITT | To evaluate metformin, and glyburide as initial monotherapies for recently diagnosed T2DM | 4360/4351 | S | Glucophage, Bristol-Myers Squibb | 2000mg/2/d | 1460 | IR | Metformin IR | gluburide/sulfonylurea derivatives | 57.2±10.1 | | 1700/58.7 | 32.1±6.1 | | 32.2±6.3 | |
| Lim et al., 2016 [22] | ITT | To investigate the efficacy and safety of initial gemigliptin or metformin monotherapy in patients with T2DM (T2D). | 433/433 | S | nd | 1868.2mg/nd | 168 | IR | Metformin IR | gemigliptin/DPP4 inhibitor | 53.7±11.2 | | 169/58.7 | 25.8±3,5 | | 26.1/3.5 | |
| Ma et al., 2014 [23] | ITT | To compare the effects of metformin or repaglinide on the fasting plasma glucose (FPG) and glycated haemoglobin (HbA1c) in newly diagnosed T2DM in China. | 107/107 | S | nd | 1500mg/nd | 90 | IR | Metformin IR | repaglinide/other | 57.1±1.6 | | 61/57 | 26.15±0.46 | | 24.45/0.33 | |
| Moses et al., 1999 [24] | ITT | To compare the effect of repaglinide or metformin monotherapy of each drug on glycemic control in patients with T2DM. | 83/82 | P/4.1years/1.8g/d | nd | 1800mg/nd | 150 | IR | Metformin IR | repaglinide/other | 59.1±8.7 | | 32/58 | 31.8±6 | | 31.3/7.2 | |
| Pavo et al., 2003 [25] | ITT | To compare the effect of pioglitazone or metformin treatment on glycemic control, as defined by change in haemoglobin A1C | 205/205 | S | nd | 2292mg/3/d | 224 | IR | Metformin IR | Pioglitazone/PPRγ receptor agonist | 55±8.8 | | 102/49.8 | 31.1±4.4 | | 31.3/4.2 | |
| Perez et al., 2009 [26] | ITT | To examine the efficacy and safety of of pioglitazone 15 mg or metformin 850 mg monotherapies in a twice-daily regimen over 24 weeks of treatment in T2DM patients who were currently not receiving antidiabetic therapy. | 600/600 | S | nd | 1700mg/2/d | 168 | IR | Metformin IR | pioglitazone/PPRγ receptor agonist | 53.8±12 | | 164/41.1 | 30.8±5.7 | | 31.2/5.5 | |
| Pratley et al., 2014a [27] | ITT | To evaluate the efficacy and safety of metformin initial therapy in drug‐naïve T2DM patients. | 784/768 | S | nd | 1000mg/2/d | 182 | IR | Metformin IR | PBO | 53.9±9.9 | | 102/45.7 | 30.2±4.84 | | 31.2/5.27 | |
| Pratley et al., 2014b |  |  |  |  |  | 2000mg/2/d |  |  | Metformin IR |  | 52.8±10.5 | | 106/48.2 | 30.5±5 | |  |  |
| Rosenstock et al., 2006 [28] | ITT | To asses the efficacy and safety of rosiglitazone or metformin monotherapies as initial therapy in patients with uncontrolled T2DM after 32 weeks of treatment. | 468/468 | S | nd | 1847mg/nd | 224 | IR | Metformin IR | Rosiglitazone/PPRγ receptor agonist | 51±10.3 | | 180/57.5 | 32.5±7 | | 32.8/7.1 | |
| Rosnstock et al., 2016 [29] | ITT | To asses the efficacy/safety of canagliflozin (CANA), a sodium–glucose cotransporter 2 (SGLT2) inhibitor, or metformin extended-release (MET) initial monotherapies in drug-naïve T2DM. | 1186/1186 | S | nd | 1930mg/1/d | 182 | XR | Metformin XR | Canagliflozin 100 mg/SGLT2 inhibitor | 54.6±10.3 | | 221/46.62 | 33±6 | | 32.4/5.4 | |
| Russell-Jones et al., 2012 [30] | ITT | To test the safety and efficacy of metformin (MET) or pioglitazone (PIO) over 26 weeks, in suboptimally treated (diet and exercise) drug-naive patients with T2DM. | 820/820 | S | nd | 2000mg/nd | 182 | IR | Metformin IR | Pioglitazone/PPRγ receptor agonist | 54.4±11 | | 251/61.4 | 30.7±5.5 | | 31.1/5.3 | |
| Schernthaner et al., 2004 [31] | ITT | To compare metabolic control in drug-naive T2DM patients given either pioglitazone or metformin. | 1199/1194 | S | nd | 2124mg/3/d | 364 | IR | Metformin IR | Pioglitazone/PPRγ receptor agonist | 56.5±9.4 | | 659/55.2 | 31.4±5.2 | | 31.2/4.9 | |
| Schweitzer et al., 2007 [32] | ITT | To evaluate the ability of vildagliptin and metformin to sustain reductions in HbA1c over a 1‐year treatment period in drug‐naïve patients with T2DM (Type 2 DM). | 780/771 | S | nd | 1988m/nd | 364 | IR | Metformin IR | Vildagliptin/DPP4 inhibitor | 53.1±11.2 | | 424/54.4 | 32.5±5.7 | | 32.4/5.7 | |
| Schweitzer et al., 2009 [33] | ITT | To evaluate the efficacy and tolerability of the dipeptidyl peptidase‐4 inhibitor, vildagliptin, and metformin in drug‐naïve elderly patients with T2DM. | 335/332 | S | nd | 1500mg/2/d | 168 | IR | Metformin IR | Vildagliptin/DPP4 inhibitor | 70.9±5.5 | | 163/48.7 | 29.4±4.6 | | 29.8/4.4 | |
| Sun et al., 2016 [34] | ITT | To compare the efficacy of acarbose and metformin in overweight and/or obese patients with newly diagnosed T2DM (T2DM). | 108/108 | S | nd | 1500mg/nd | 168 | IR | Metformin IR | Acarbose/Other | 52.5±7.1 | | 57/52.8 | 27.02±1.85 | | 27.07/1.97 | |
| Takeda et al., 2016 [35] | ITT | To evaluate the efficacy and safety of metformin alone on T2DM (T2DM). | 647/647 | S | Glucophage | 1000mg/2/d | 182 | IR | Metformin IR | PBO | 52.9±10.1 | | 177/54.5 | 26.3±3.6 | | 26.6/4.2 | |
| Takeshita et al., 2019 [36] | PP | To investigate the effects of metformin or a dipeptidyl peptidase‐4 inhibitor, alogliptin, on body composition in a 12‐week randomized trial in Japanese participants with T2DM. | 84/80 | S | Metformin (Sumitomo Dainippon Pharma Co. Ltd., Osaka, Japan) | 1076mg/2/d | 84 | IR | Metformin IR | alogliptin/DPP4 inhibitor | 63.4±11.8 | | 58/69 | 23.8±4 | | 25.4±6 | |
| Umpierrez et al., 2014 [37] | ITT | To compare the efficacy and safety of monotherapy with dulaglutide, a once-weekly GLP-1 receptor agonist, to metformin-treated patients with T2DM at 26 weeks. | 807/807 | S | nd | 2000mg/nd | 264 | IR | Metformin IR | dulaglutide 0,75 mg/GLP-1 receptor agonist | 55.5±10.5 | | 239/44.4 | 33±5 | | 33±6 | |
| Williams -Herman et al., 2010a [38] | ITT | To assess the 104‐week efficacy and safety of sitagliptin and metformin as monotherapies in patients with T2DM and inadequate glycaemic control (HbA1c 7.5–11%) on diet and exercise. | 1091/1091 | S | nd | 1000mg/2/d | 728 | IR | Metformin IR | Sitagliptin 100 mg/DPP4 | 53.4±10.2 | | 182/50.4 | 32.1±6.8 | | 31.2/5.9 | |
| Williams -Herman et al., 2010b |  |  |  |  |  | 2000mg/2/d |  |  | Metformin IR |  | 53.2±9.9 | | 175/48.5 | 32.2±7.1 | |  |  |
| Yoon et al., 2010 [39] | ITT | To evaluate the efficacy of glimepiride or metformin as initial treatment for drug-naïve T2DMmellitus patients | 349/349 | S | nd | 1238mg/2/d | 336 | IR | Metformin IR | glimepiride/sulfonylurea derivatives | 51.29±8.72 | | 132/56.9 | 25.7±3.2 | | 25.5/3.1 | |
| Yuan et al., 2012 [40] | ITT | To evaluate the efficacy and tolerability of exenatide or metformin monotherapy in obese patients with T2DM. | 59/59 | S | nd | 2000mg/2/d | 182 | IR | Metformin IR | Exenatide/GLP-1 receptor agonist | 57.8±9.4 | | 29/49.2 | 29.3±2.6 | | 30.6/2.8 | |
| List et al., 2009 [41] | ITT | To evaluate the safety and efficacy of metformin in type 2 diabetic patients. | 389/389 | S | nd | 1500mg/nd | 84 | XR | Metformin XR | PBO | 53.5±10 | | 57/51.8 | 32±5 | | 32/5 | |
| NCT00859898, 2014 [42] | ITT | To compare the change from baseline in haemoglobin A1C achieved with metformin XR monotherapy and compared with Dapagliflozin monotherapy, after 24 weeks of oral administration of double-blind treatment. | 638/638 | S | Glucophage® | 2000mg/1/d | 168 | XR | Metformin XR | Dapagliflozin/SGLT2 inhibitor | 51.9±11 | | 202/47.3 | < 25: 21; <27: 20; <30: 49 ≥30: 118 | | < 25: 25; <27: 24; <30: 39  ≥30: 131 | |

Abbreviations: MET - metformin ; ITT - intention to treat; PP - per protocol; S - started metformin therapy in the study; P - preexisting metformin therapy; nd – no data.

ESM Table S2. Included studies comparing different metformin formulations

| **Study characteristics** | | | | **MET treatment** | | | | | **comparator** | **Patients characteristics** | | BMI (kg/m2) at baseline | |
| --- | --- | --- | --- | --- | --- | --- | --- | --- | --- | --- | --- | --- | --- |
| **Reference** | **ITT/PP** | **Focus of study** | **N total randomized/analyzed** | **MET (P/S)/p dose** | **MET commercial labeling** | **Dosage (unit/day)** | **Duration (days)** | **MET XR/IR** |  | **age (Ø ±SD)** | **Male (N/%)** | **MetforminØ±SD** | **Control Ø±SD** |
| Aggarwal et al., 2017 [43] | ITT | To compare the efficacy and safety of XR and IR metformin in T2DM pts | 568/568 | s/na/na | Glucophage® XR, Bristol- Myers Squibb | 1920.5mg/day | 168 | XR | MET-IR/ MET XR/MET IR | 56±10.5 | 295/54.7 | 32.9±5.5 | 32.8±5.4 |
| DeFronzo et al., 2016a [44] | ITT | To compare the bioavailability and effects on circulating glucose and gut hormones (glucagon-like peptide-1, peptide YY) of Metformin DR dosed twice-daily to twice-daily immediate-release metformin (Metformin IR) | 24/22 | S | nd | 2000mg/2/d | 5 | XR | MET-IR 1000 mg/ MET XR/MET IR | 51.3±10 | 12\|50 | 33.3±4.1 | 33.3±4.1 |
| DeFronzo et al., 2016b |  |  | 24/22 |  | nd | 1000mg/2/d |  |  | MET-IR 1000 mg/ MET XR/MET IR | 51.3±10 | 12\|50 | 33.3±4.1 | 33.3±4.1 |
| DeFronzo et al., 2016c |  |  | 26/23 |  | nd | 1000mg/1/d | 7 |  | MET-DR 500 mg twice daily/ MET DR/MET XR | 50.9±10.9 | 10\|38 | 31.5±3.2 | 31.5±3.2 |
| DeFronzo et al., 2016d |  |  | 26/24 |  | nd | 1000mg/2/d |  |  | MET-DR 500 mg twice daily/ MET DR/MET XR | 50.9±10.9 | 10\|38 | 31.5±3.2 | 31.5±3.2 |
| Derosa et al., 2017a [45] | nd | To evaluate the effects of IR and XR metformin on the gastrointestinal tolerability and glycemic control | 253/nd | S | nd | 1000mg/1/d | 180 | XR | Metformin IR/MET XR/MET IR | 56.2±11 | 122/48.2 | 27.5±2.4 | 27.3±2.1 |
| Derosa et al., 2017b |  |  |  |  |  |  |  |  | Metformin IR/MET XR/MET IR |  |  |  |  |
| Fujioka et al., 2003a [46] | ITT | To determine the effects on glycemic control of a switch to MXR therapy in patients with T2DM currently treated with MIR | 217/217 | P/≥10 weeks/1g/d | Glucophage ® XR | 1000mg/1/d | 168 | XR | Metformin-IR/ MET XR/MET IR | 54±nd | 65/44.5 | 32±nd | 33±nd |
| Fujioka et al., 2003b |  |  |  |  |  | 1500mg/1/d |  |  |  | 54.5±nd | 59/41.5 | 31±nd | 33±nd |
| Gao et al., 2008 [47] | PP | To investigate the effects of XR and IR metformin on post‐prandial glycaemic excursion, chronic glycaemia, lipid profiles, insulin resistance and islet function in T2DM. | 150/140 | P/2 weeks/1.5g/d | Glucophage® | 1500mg/1/d | 84 | XR | Metformin-IR/ MET XR/MET IR | 54.6±8.5 | 75/50 | 26.4±3.1 | 26.2±2.9 |
| Ji et al., 2017 [48] | ITT | To investigate whether once‐daily metformin extended release (XR) is superior in terms of GI tolerability, with non‐inferior efficacy, compared with thrice‐daily metformin immediate release (IR) in treatment‐naïve Chinese patients with T2DM. | 532/525 | S | nd | 1988mg/1/d | 112 | XR | Metformin IR/MET XR/MET IR | 53.±10 | 312/58.6 | 26.2±3.2 | 26.2±3.1 |
| Schwartz et al., 2006a [49] | ITT | To determine the efficacy and safety of a novel extended-release metformin in patients with T2DM. | 750/706 | S | Glumetza | 1500mg/1/d | 168 | XR | Metformin IR (AM/PM)/ MET XR/MET IR | 54±12 | 178/50.6 | 33.4±6.6 | 33.8/6.8 |
| Schwartz et al., 2006b |  |  |  |  |  | 1500mg/2/d |  |  |  | 54±12.1 | 206/57.9 | 33±6.3 |  |
| Schwartz et al., 2006c |  |  |  |  |  | 2000mg/1/d |  |  |  | 54.5±12.1 | 186/53.8 | 33.7±6.6 |  |

Abbreviations: MET - metformin ; ITT - intention to treat; PP - per protocol; S - started metformin therapy in the study; P - preexisting metformin therapy; nd – no data.

ESM Table S3. Included studies comparing metformin and combination of metformin with another antidiabetic drug

| **Study characteristics** | | | | **MET treatment** | | | | | **comparator** | **Patients characteristics** | | BMI (kg/m2) at baseline | |
| --- | --- | --- | --- | --- | --- | --- | --- | --- | --- | --- | --- | --- | --- |
| **Reference** | **ITT/PP** | **Focus of study** | **N total randomized/analyzed** | **MET (P/S)/p dose** | **MET commercial labeling** | **Dosage (unit/day)** | **Duration (days)** | **MET XR/IR** |  | **age (Ø ±SD)** | **Male (N/%)** | **MetforminØ±SD** | **Control Ø±SD** |
| Bailey et al., 2005 [50] | ITT | To investigate the benefits of fixed-dose combination rosiglitazone and MET (RSG/MET) compared with high-dose MET monotherapy in patients with type 2 DM | 569/568 | P/>8 weeks/2g/d | nd | 3000mg/nd | 168 | IR | fixed-dose combination rosiglitazone and MET/MET/MET+add on | 57.9±8.3 | 327/57.6 | 32,1±4.9 | 32.2±4,8 |
| Fonseca et al., 2011 [51] | ITT | To investigate whether patients taking metformin for T2DM (T2DM) have improved glycaemic control without compromising tolerability by adding an agent with a complementary mechanism of action vs. up titrating metformin. | 282/282 | P/≥8 weeks/1,5g/d | Glucophage XR | 2000mg/1/d | 126 | XR | saxagliptin + metformin XR/MET/MET+add on | 55.4±9.7 | 130/46.1 | 31±10.6 | 30.8±5 |
| Gao et al., 2020 [52] | ITT | To investigate the therapeutic effects of combination of linagliptin and metformin on treatment of elderly T2DM patients and its influences on serum uric acid, insulin resistance and insulin â cell function | 200/200 | S | nd | 1500mg/3/d | 90 | IR | Metformin + linagliptin/ MET/MET+add on | 70.3±4.1 | 97/48.5 | 26.89±2.99 | 27.03±3.01 |
| Ghosh et al., 2014 [53] | ITT | To compare the effectiveness and safety of add on therapy of bromocriptine with metformin in T2DM (DM) patients. | 74/74 | S | nd | 1000mg/2/d | 84 | XR | metformin 500 mg b.i.d. + bromocriptine 0,8 q.d./MET/MET+add on | 50.4±nd | nd/nd | nd±nd | nd±nd |
| Gottschalk et al., 2007 [54] | ITT | To compare the efficacy and safety of glimepiride versus metformin in paediatric subjects with T2DM inadequately controlled with diet and exercise alone or oral monotherapy. | 285/284 | S | nd | 1408mg/2d | 168 | IR | glimepiride/sulfonylurea derivatives | 13.8±2.3 | 88/33.5 | 31.6±8.17 | 31.57/8.48 |
| Hemans et al., 2012 [55] | ITT | To compere efficacy and tolerability of two treatment intensification strategies: adding saxagliptin or uptitrating metformin monotherapy, in patients with T2DM (T2D) and inadequate glycaemic control on a sub-maximal metformin dose. | 286/286 | P/≥12 weeks/1,5g/d | nd | 2404mg/nd | 168 | IR | saxagliptin + metformin/MET/MET+add on | 58.7±10.6 | 164/57.3 | 31.2±5.7 | 32.1±6.7 |
| Ji et al., 2015 [56] | ITT | To investigate the efficacy and safety of linagliptin ? low-dose (LD) metformin once daily versus high-dose (HD) metformin twice daily in treatment-native patients with T2DM. | 689/639 | S | nd | 1798mg/2/d | 98 | IR | linagliptin + metformin/ MET/MET+add on | 53±10.7 | 327/47.5 | 29±5.6 | 29±5.7 |
| Kim et al., 2014 [57] | ITT | To compare the efficacy and safety of early combination therapYwith glimepiride/metformin to metformin uptitration in reducing glycated hemoglobin (HbA1c) levels in Korean type 2 diabetic patients inadequately controlled on low-dose metformin monotherapy | 209/208 | P≥4/1g/d | nd | 1314mg/2/d | 182 | IR | Glimepiride + metformin/ MET/MET+add on | 55.7±9,1 | 103/49.3 | 25.7±3.2 | 25.5±3.5 |
| Nauck et al., 2012 [58] | ITT | To investigate efficacy and safety of dual therapy with liraglutide and metformin in comparison to glimepiride and metformin, and metformin monotherapy over 2 years in patients with T2DM. | 1091/1087 | P≥3months/nd | nd | 2000mg/2/d | 730 | IR | Metformin IR + Glimepiride/MET/MET+add on | 56.7±9 | 212/57.9 | 31.6±4.4 | 31.2/4.6 |
| Nauck et al., 2008 [59] | ITT | To evaluate the efficacy and safety of alogliptin, a new dipeptidyl peptidase‐4 inhibitor, for 26 weeks at once‐daily doses of 12.5 and 25 mg in combination with metformin in patients whose HbA1c levels were inadequately controlled on metformin alone. | 527/527 | P≥3months≥1500 mg per day for at least 8 weeks | nd | 1868mg/nd | 182 | IR | Metformin + Alogliptin 12,5 mg/MET/MET+add on | 55.3±11 | 151/47.6 | 32±6 | 32/5 |
| Neutel et al., 2013 [60] | ITT | To evaluate the impact of saxagliptin added to metformin extended release (XR) and uptitrated metformin XR on daily glucose measurements and their tolerability in patients with T2DM (T2DM) inadequately controlled with metformin monotherapy. | 93/93 | P≥12/1.5g/d | nd | 2000mg/1/d | 28 | XR | Metformin XR + saxagliptin/MET/MET+add on | 52.2±9.7 | 47/50.5 | 31.1±4.3 | 32.5/5.1 |
| Matthews et al., 2019 [61] | ITT | To compare early combination therapy of metformin plus dipeptidyl peptidase-4 inhibitor vildagliptin with standard-of-care metformin monotherapy, | 2001/1999 | P/3/1g/d | nd | 1500mg/2/d | 182 | IR | Metformin + vildagliptin/MET/MET+add on | 54.4±9.4 | 941/47 | 31±4.7 | 31.2/4.8 |
| Oh et al., 2019 [62] | ITT | To compare the efficacy and safety of a fixed-dose combination of voglibose plus metformin (vogmet) with metformin monotherapy in drug-naive newly-diagnosed T2DM. | 187/172 | S | nd | 1323mg/3/d | 168 | IR | Metformin + voglibose/MET/MET+add on | 52.6±8.8 | 99/57.6 | 25.1±2.5 | 24.5/2.4 |
| Reasner et al., 2011 [63] | ITT | To compare the glycaemic efficacy and safety of initial combination therapy with the fixed‐dose combination of sitagliptin and metformin versus metformin monotherapy in drug‐naive patients with T2DM. | 1250/1246 | S | nd | 2000mg/2/d | 126 | IR | Sitagliptin and metformin/MET/MET+add on | 49.7±10.5 | 709/56.9 | 33.7±7.8 | 32.9/7.2 |
| Rosenstock et al., 2010a [64] | ITT | The efficacy and safety of the 11βHSD1 inhibitor INCB13739 were assessed when added to ongoing metformin monotherapy in patients with T2DM exhibiting inadequate glycemic control (A1C 7–11%). | 302/302 | P≥10/1.5g/d | nd | 1500mg/nd | 84 | IR | Metformin + 11-β-Hydroxysteroid Dehydrogenase Type 1 Inhibitor INCB13739 5 mg/MET/MET+add on | 53.5±10.2 | 55/54.5 | 33±5 | 33/6 |
| Rosenstock et al., 2010b [65] | ITT | To evaluate the efficacy and safety of initial  combination therapy with metformin plus colesevelam in  patients with early type 2 diabetes. | 286/286 | S | nd | 1700mg/2/d | 112 | IR | Metformin + colesevelam/MET/MET+add on | 53.±10.8 | 125/43.7 | 29.8±4.4 | 30,6/4.7 |
| Seino et al., 2012 [66] | ITT | To evaluate the efficacy and safety of alogliptin added to metformin versus metformin monotherapy in Japanese patients with T2DM who achieved inadequate glycaemic control on metformin (500 or 750 mg/day) + diet/exercise. | 288/288 | P/12weeks/500-750mg/d | Glycoran | 750mg/3/d | 84 | IR | Metformin IR + alogliptin 12.5 mg/MET/MET+add on | 52.7±8.4 | 132/68.8 | 26.14±4.58 | 25.63/4.1 |
| Stewart et al., 2006 [67] | ITT | To investigate the effect of metformin plus roziglitazione (RSGMET) compared with metformin alone (MET) on glycaemic control in well‐controlled T2DM. | 526/526 | S | nd | 2628mg/nd | 224 | IR | Metformin IR + roziglitazone/MET/MET+add on | 59±8.1 | 290/55.1 | 30.6±5.5 | 30.9/5.4 |
| Weissman et al., 2005 [68] | ITT | To compare the efficacy, safety and tolerability of rosiglitazone (RSG) added to submaximal doses of metformin (MET) with dose escalation to the maximal effective dose of MET monotherapy in T2DM. | 766/766 | P>3months≤1000mg/day | nd | 2000mg/2/d | 168 | IR | Rosiglitazone + metformin/MET/MET+add on | 55.6±10.7 | nd/nd | 33.8±6.5 | 34.4±7.3 |
| Yang et al., 2011 [69] | ITT | To assess efficacy and safety of saxagliptin added to metformin versus placebo plus metformin in Asian patients with T2DM (T2DM) and inadequate glycemic control on metformin alone | 570/570 | P≥8weeks/1.6g/d | nd | 1606mg/nd | 168 | IR | saxagliptin + metformin/MET/MET+add on | 54.1±10.3 | 275/48.2 | 26.1±3.5 | 26.3/3.6 |
| Yuxin et al., 2019a [70] | ITT | To compare gastrointestinal adverse events resulting from different doses of metformin used for the treatment of elderly people with type 2 diabetes. | 361/361 | S | Glucophage | 1500mg/3/d | 84 | IR | Metformin IR 1g/d/MET HD/MET LD | 69±6.6 | 136/56.4 | 25.1±4 | 25.3/3.3 |
| Yuxin et al., 2019b |  |  |  |  |  | 2000mg/4/d |  |  |  | 68.5±6.2 | 140/58.3 | 26.2±3.1 |  |
| Zack et al., 2014a [71] | ITT | To explore the potential for a drug–drug interactionin two phase 1 clinical studies in subjects with T2DM to evaluate the pharmacokinetics and safety of metformin 1000 mg BID when administered with ranolazine 1000 mg BID (Study 1, N = 28) as compared to metformin alone. | 28/28 | P≥4 weeks/850-1000mg/2/d | nd | 2000mg/2/d | 20 | IR | Metformin 1000mg BID + Ranolazin 1000mg BID/MET/MET+add on | 57,1±42-65 | 15/53.6 | 31.4±nd | 31.4/nd |
| Zack et al., 2014b | ITT | To explore the potential for a drug–drug interaction in two phase 1 clinical studies in subjects with T2DM to evaluate the pharmacokinetics and safety of metformin 1000 mg BID when administered with ranolazine 500 mg BID (Study 2, N = 25) as compared to metformin alone. | 24/24 | S≥4weeks≥1500mg/1/d | nd | 2000mg/2/d | 5 | IR | Metformin 1000mg BID + Ranolazin 1000mg BI/MET/MET+add on | 56.5±44-65 | 13/52 | 30.9±nd | 30.9/nd |

Abbreviations: MET - metformin ; ITT - intention to treat; PP - per protocol; S - started metformin therapy in the study; P - preexisting metformin therapy; nd – no data.

ESM Table S4. GI complications in persons receiving metformin

| **Reference** | **Discontinuation due to adverse events** | | | **abdominal pain (n)** | | **Diarrhea (n)** | | **Vomiting (n)** | | **Bloating (n)** | | **Nausea (n)** | | **Constipation (n)** | |
| --- | --- | --- | --- | --- | --- | --- | --- | --- | --- | --- | --- | --- | --- | --- | --- |
|  | **MET events/ total** | **CTRL events/ total** | **type of adverse event** | **MET cases/ total** | **CTRL cases/ total** | **MET cases/ total** | **CTRL cases/ total** | **MET cases/ total** | **CTRL cases/ total** | **MET cases/ total** | **CTRL cases/ total** | **MET cases/ total** | **CTRL cases/ total** | **MET cases/ total** | **CTRL cases/ total** |
| Aggarwal et al., 2017 | 6/268 | 1/271 | most frequently GI disorders | 4/283 | 6/285 | 25/283 | 22/285 | 7/283 | 4/285 | nd/nd | nd/nd | 13/283 | 8/285 | nd/nd | nd/nd |
| Aschner et al.., 2010 | 19/522 | 0/528 | nd | 20/522 | 11/528 | 57/522 | 19/528 | 7/522 | 2/528 | nd/nd | nd/nd | 16/522 | 6/528 | 5/522 | 9/528 |
| Bailey et al., 2005 | 22/280 | 12/288 | most frequently GI disorders | 25/280 | 17/288 | 39/280 | 17/288 | nd/nd | nd/nd | nd/nd | nd/nd | nd/nd | nd/nd | nd/nd | nd/nd |
| Blonde et al., 2002 | 8/153 | 5/164 | nd | 13/153 | 4/164 | 38/153 | 10/164 | 19/153 | 9/164 | 3/153 | 0/164 | 19/153 | 9/164 | nd/nd | nd/nd |
| Ferrannini et al.., 2013 | 1\|56 | 5/106 | genital infection led to most discontinuations in comparator arm | nd/nd | nd/nd | 3\|56 | 2/106 | nd/nd | nd/nd | nd/nd | nd/nd | 3\|56 | 2/106 | nd/nd | nd/nd |
| Bosi et al., 2009 | 13/292 | 7/297 | most commonly diarrhoea, headache, nasopharyngitis | 10/292 | 6/297 | 32/297 | 7/297 | 7/292 | 1/297 | nd/nd | nd/nd | 17/292 | 7/297 | 5/297 | 10/297 |
| DeFronzo et al., 2016a | 0/24 | 2\|24 | vomiting | nd/nd | nd/nd | 3\|20 | 3\|22 | 0/20 | 2\|22 | nd/nd | nd/nd | 0/20 | 2\|22 | nd/nd | nd/nd |
| DeFronzo et al., 2016b | 0/24 | 2\|24 | vomiting | nd/nd | nd/nd | 2\|20 | 3\|22 | 0/20 | 2\|22 | nd/nd | nd/nd | 0/20 | 2\|22 | nd/nd | nd/nd |
| DeFronzo et al., 2016c | nd/nd | nd/nd | nd | nd/nd | nd/nd | 2\|23 | 1\|23 | 0/23 | 2\|23 | nd/nd | nd/nd | 0/23 | 2\|23 | nd/nd | nd/nd |
| DeFronzo et al., 2016d | nd/nd | nd/nd | nd | nd/nd | nd/nd | 0/24 | 1\|23 | 0/24 | 2\|23 | nd/nd | nd/nd | 0/24 | 2\|23 | nd/nd | nd/nd |
| DeFronzo et al., 1995 | 14/143 | 2/146 | most frequently GI disorders (11/14 in metformin arm) | nd/nd | nd/nd | 11/143 | nd/nd | nd/nd | nd/nd | nd/nd | nd/nd | 6/143 | nd/nd | nd/nd | nd/nd |
| Del Prato et al., 2002 | 16/284 | 6/144 | most frequently GI disorders | nd/nd | nd/nd | 40/143 | 2/144 | nd/nd | nd/nd | nd/nd | nd/nd | nd/nd | nd/nd | nd/nd | nd/nd |
| Derosa et al., 2017a | nd/nd | nd/nd | nd | nd/nd | nd/nd | 2/124 | 8/119 | 1/124 | 5/119 | 3/124 | 29/119 | 2/124 | 3/119 | nd/nd | nd/nd |
| Derosa et al., 2017b | nd/nd | nd/nd | nd | nd/nd | nd/nd | 0/120 | 7/115 | 0/120 | 3/115 | 5/120 | 22/115 | 2/120 | 2/115 | nd/nd | nd/nd |
| Dou et al., 2017 | 4/210 | 5/214 | nd | 4/210 | 4/214 | 13/210 | 6/214 | nd/nd | nd/nd | 3/210 | 5/214 | nd/nd | nd/nd | nd/nd | nd/nd |
| Henry et al., 2018a | 3\|94 | 6\|96 | most frequently GI disorders (diarrhea, nausea, and abdominal pain) | nd/nd | nd/nd | 7\|94 | 0/96 | nd/nd | nd/nd | nd/nd | nd/nd | nd/nd | nd/nd | nd/nd | nd/nd |
| Henry et al., 2018b | 2\|95 | 6\|96 |  | nd/nd | nd/nd | 7\|95 | 0/96 | nd/nd | nd/nd | nd/nd | nd/nd | 3\|95 | 1\|96 | nd/nd | nd/nd |
| Henry et al., 2018c | 4\|96 | 6\|96 |  | nd/nd | nd/nd | 11\|96 | 0/96 | nd/nd | nd/nd | nd/nd | nd/nd | 1\|96 | 1\|/96 | nd/nd | nd/nd |
| Henry et al., 2018d | 2\|96 | 6\|96 |  | nd/nd | nd/nd | 11\|96 | 0\|96 | nd/nd | nd/nd | nd/nd | nd/nd | 3\|96 | 1\|96 | nd/nd | nd/nd |
| Henry et al., 2018e | 8\|94 | 6\|96 | most frequently GI disorders (diarrhea, nausea, and abdominal pain) | nd/nd | nd/nd | 13/94 | 0/96 | nd/nd | nd/nd | nd/nd | nd/nd | 9\|94 | 1\|96 | nd/nd | nd/nd |
| Feinglos et al., 2005 | 2\|34 | 2\|37 | none were GI disorders (hyperglycaemia, cerebrovascular disorder, palpitation, hyper- triglyceridemia and diplopia) | nd/nd | nd/nd | nd/nd | nd/nd | 1\|34 | 4/176 | nd/nd | nd/nd | 2\|34 | 7\|176 | nd/nd | nd/nd |
| Fonseca et al., 2011 | 7/144 | 1/138 | Four gastrointestinal adverse events resulted in discontinuation from the uptitrated metformin XR group | nd/nd | nd/nd | 5/144 | 8/138 | 0/144 | 3/138 | 56/144 | 63/138 | 3/144 | 3/138 | nd/nd | nd/nd |
| Fuanhchan et al., 2011 | 0/33 | 0/33 | few patients discontinued metformin XR treatment for GI disorders | 2\|33 | 1\|32 | 1\|33 | 1\|32 | nd/nd | nd/nd | 4\|33 | 3\|32 | 4\|33 | 2\|32 | 2\|33 | 2\|32 |
| Fujioka et al., 2005a | 8/161 | 2\|79 | few patients discontinued metformin XR treatment for GI disorders | 12/159 | 4\|79 | 11/159 | 4\|79 | 15/159 | 3\|79 | nd/nd | nd/nd | 15/159 | 3\|79 | nd/nd | nd/nd |
| Fujioka et al., 2005b | 4/128 | 1/117 |  | 7/117 | 3/116 | 6/117 | 4/116 | 5/117 | 2/116 | nd/nd | nd/nd | 5/117 | 2/116 | nd/nd | nd/nd |
| Fujioka et al., 2005c | 3/120 | 1/117 |  | 7/119 | 3/116 | 15/119 | 4/116 | 11/119 | 2/116 | nd/nd | nd/nd | 11/119 | 2/116 | nd/nd | nd/nd |
| Fujioka et al., 2005d | 5/120 | 1/117 |  | 6/120 | 3/116 | 22/120 | 4/116 | 14/120 | 2/116 | nd/nd | nd/nd | 14/120 | 2/116 | nd/nd | nd/nd |
| Fujioka et al., 2005e | 4/134 | 1/117 |  | 4/133 | 3/116 | 20/133 | 4/116 | 12/133 | 2/116 | nd/nd | nd/nd | 12/133 | 2/116 | nd/nd | nd/nd |
| Fujioka et al., 2005f | 1/123 | 1/117 |  | 8/133 | 3/116 | 17/133 | 4/116 | 9/133 | 2/116 | nd/nd | nd/nd | 9/133 | 2/116 | nd/nd | nd/nd |
| Fujioka et al., 2003a | 6\|75 | 1\|71 | Three due to GI disorders | 3\|75 | 1\|71 | 4\|75 | 2\|71 | 2\|75 | 3\|71 | 3\|75 | 1\|71 | 2\|75 | 3\|71 | nd/nd | nd/nd |
| Fujioka et al., 2003b | 1\|71 | 1\|71 | diarrhea | 1\|71 | 1\|71 | 11\|71 | 2\|71 | 2\|71 | 3\|71 | 2\|71 | 1\|71 | 2\|71 | 3\|71 | nd/nd | nd/nd |
| Gao et al., 2008 | 0/75 | 0/75 | nd | 2\|69 | 2\|71 | 0/69 | 4\|71 | nd/nd | nd/nd | 1\|69 | 1\|71 | 0\|69 | 1\|71 | nd/nd | nd/nd |
| Gao et al., 2020 | nd/nd | nd/nd | nd | 9/100 | 6/100 | 9/100 | 6/100 | 14/100 | 9/100 | nd/nd | nd/nd | 14/100 | 9/100 | 13/100 | 7/100 |
| Garber et al., 1997a | 3\|73 | 5\|79 | usually digestive disturbances | 2\|73 | 0/79 | 6\|73 | 4\|79 | nd/nd | nd/nd | nd/nd | nd/nd | 5\|73 | 4\|79 | nd/nd | nd/nd |
| Garber et al., 1997b | 4\|73 | 5\|79 |  | 1\|73 | 0/79 | 15/73 | 4\|79 | nd/nd | nd/nd | nd/nd | nd/nd | 7\|73 | 4\|79 | nd/nd | nd/nd |
| Garber et al., 1997c | 5\|76 | 5\79 |  | 3\|76 | 0/79 | 9\|76 | 4\|79 | nd/nd | nd/nd | nd/nd | nd/nd | 6\|76 | 4\|79 | nd/nd | nd/nd |
| Garber et al., 1997d | 5\|73 | 5\|79 |  | 0\|73 | 0/79 | 14/73 | 4\|79 | nd/nd | nd/nd | nd/nd | nd/nd | 1\|73 | 4\|79 | nd/nd | nd/nd |
| Garber et al., 1997e | 11\|77 | 5\|79 |  | 2\|77 | 0/79 | 11\|77 | 4\|79 | nd/nd | nd/nd | nd/nd | nd/nd | 9\|77 | 4\|79 | nd/nd | nd/nd |
| Garber et al., 2003 | nd/nd | nd/nd | The most common reasons for discontinuation was adverse events other than hypoglycemia (3.5%). | nd/nd | nd/nd | nd/nd | nd/nd | nd/nd | nd/nd | nd/nd | nd/nd | 17/164 | 10/151 | nd/nd | nd/nd |
| Ghosh et al., 2014 | 0/23 | 2\|25 | nd | nd/nd | nd/nd | nd/nd | nd/nd | 0/23 | 3\|25 | nd/nd | nd/nd | 0/23 | 3\|25 | nd/nd | nd/nd |
| Goke et al., 2008 | 3/158 | 5/304 | nd | 11/158 | 7/304 | 45/158 | 19/304 | nd/nd | nd/nd | 8/158 | 3/304 | 15/158 | 9/304 | nd/nd | nd/nd |
| Goldstein et al., 2003 | 1\|75 | 3\|84 | only GI disorders | 5\|75 | 7\|84 | 13\|75 | 11\|84 | 6\|75 | 5\|84 | nd/nd | nd/nd | 6\|75 | 5\|84 | nd/nd | nd/nd |
| Gottschalk et al., 2007 | 1/142 | 1/142 | nd | 2/131 | 2/132 | 6/131 | 1/132 | nd/nd | nd/nd | nd/nd | nd/nd | 4/131 | 1/132 | nd/nd | nd/nd |
| Haak et al., 2012a | 3/144 | 5\|72 | nd | nd/nd | nd/nd | 3/144 | 2\|72 | 0/144 | 1\|72 | nd/nd | nd/nd | 0/144 | 0/72 | 3/144 | 1\|72 |
| Haak et al., 2012b | 6/147 | 5\|72 |  | nd/nd | nd/nd | 8/147 | 2\|72 | 1/147 | 1\|72 | nd/nd | nd/nd | 5/147 | 0\|72 | 0/147 | 1\|72 |
| Henry et al., 2012a | 6/201 | 5/203 | nd | nd/nd | nd/nd | 14/201 | 8/203 | nd/nd | nd/nd | nd/nd | nd/nd | 8/201 | 4/203 | nd/nd | nd/nd |
| Henry et al., 2012b | 8/208 | 9/219 |  | nd/nd | nd/nd | 20/208 | 6/219 | nd/nd | nd/nd | nd/nd | nd/nd | 5/208 | 8/219 | nd/nd | nd/nd |
| Hemans et al., 2012 | 3/139 | 1/147 | only GI disorders | 3/139 | 1/147 | 17/139 | 9/147 | 3/139 | 3/147 | nd/nd | nd/nd | 1/139 | 4/147 | nd/nd | nd/nd |
| Horton et al., 2004 | nd/nd | nd/nd | nd | 7/104 | 5/104 | 21/104 | 7/104 | nd/nd | nd/nd | nd/nd | nd/nd | 10/104 | 4/104 | 6/104 | 3/104 |
| Jadzinsky et al., 2009 | 11/328 | 8/335 | nd | nd/nd | nd/nd | 24/328 | 10/335 | nd/nd | nd/nd | nd/nd | nd/nd | nd/nd | nd/nd | nd/nd | nd/nd |
| Ji et al., 2016a | 3/126 | 3/126 | nd | 4/126 | 2/126 | 4/126 | 4/126 | 1/126 | 0/126 | nd/nd | nd/nd | 1/126 | 0/126 | nd/nd | nd/nd |
| Ji et al., 2016b | 2/124 | 3/126 |  | 5/124 | 2/126 | 9/124 | 4/126 | 1/124 | 0/126 | nd/nd | nd/nd | 4/124 | 0/126 | nd/nd | nd/nd |
| Ji et al., 2017 | 15/265 | 19/267 | most frequently nausea and diarrhea | 5/264 | 6/261 | 33/264 | 43/261 | nd/nd | nd/nd | 17/264 | 17/261 | 12/264 | 16/261 | 5/264 | 2/261 |
| Ji et al., 2015 | 3/345 | 3/ | nd | 21/341 | 10/298 | 54/341 | 36/298 | 6/341 | 5/298 | nd/nd | nd/nd | 16/341 | 13/298 | nd/nd | nd/nd |
| Kahn et al., 2006 | 178/1454 | 215/1441 | nd | 224/1454 | 163/1441 | 345/1454 | 142/1441 | 84/1454 | 45/1441 | nd/nd | nd/nd | 170/1454 | 99/1441 | nd/nd | nd/nd |
| Kim et al., 2014 | 3/108 | 0/100 | nd | 1/108 | 4/100 | 5/108 | 1/100 | nd/nd | nd/nd | nd/nd | nd/nd | nd/nd | nd/nd | nd/nd | nd/nd |
| Lim et al., 2016 | 5/150 | 2/142 | nd | nd/nd | nd/nd | 11/150 | 0/142 | nd/nd | nd/nd | nd/nd | nd/nd | nd/nd | nd/nd | nd/nd | nd/nd |
| Ma et al., 2014 | nd/nd | nd/nd | nd | 0/54 | 0/53 | 0/54 | 0/53 | 0/54 | 0/53 | 0/54 | 0/53 | 0/54 | 0/53 | 0/54 | 0/53 |
| Moses et al., 1999 | nd/nd | nd/nd | nd | nd/nd | nd/nd | 8\|27 | 2\|28 | nd/nd | nd/nd | nd/nd | nd/nd | nd/nd | nd/nd | nd/nd | nd/nd |
| Nauck et al., 2012 | 3/122 | 14/244 | nd | 0/61 | 4/183 | 5/121 | 14/242 | 0/121 | 1/242 | 0/61 | 0/183 | 5/121 | 10/242 | lut.61 | 3/183 |
| Nauck et al., 2008 | 1/104 | 7/213 | few GI disorders | nd/nd | nd/nd | 6/104 | 6/213 | nd/nd | nd/nd | nd/nd | nd/nd | nd/nd | nd/nd | nd/nd | nd/nd |
| Neutel et al., 2013 | 0/47 | 0/46 | nd | 1\|47 | 0/46 | sty.47 | 0/46 | nd/nd | nd/nd | nd/nd | nd/nd | 1\|47 | 0/46 | nd/nd | nd/nd |
| Matthews et al., 2019 | 44/1003 | 28/998 | nd | 31/1001 | 37/998 | 104/1001 | 105/998 | 0/1001 | 1/998 | nd/nd | nd/nd | 20/1001 | 30/998 | nd/nd | nd/nd |
| Oh et al., 2019 | mar.92 | 4\|95 | nd | 6\|84 | 3\|88 | 37/84 | 17/88 | nd/nd | nd/nd | 0/84 | 1\|88 | 14/84 | 9\|88 | 4\|84 | 5\|88 |
| Pavo et al., 2003 | 0/100 | 2/105 | nd | nd/nd | nd/nd | 16/100 | 3/105 | nd/nd | nd/nd | nd/nd | nd/nd | nd/nd | nd/nd | nd/nd | nd/nd |
| Perez et al., 2009 | 15/209 | 15/190 | nd | 7/209 | 3/190 | 32/209 | 5/190 | nd/nd | nd/nd | nd/nd | nd/nd | nd/nd | nd/nd | nd/nd | nd/nd |
| Pratley et al., 2014a | 3/114 | 4/109 | most frequently GI disorders | nd/nd | nd/nd | 4/109 | 3/106 | nd/nd | nd/nd | nd/nd | nd/nd | 4/109 | 1/106 | nd/nd | nd/nd |
| Pratley et al., 2014b | 2/111 | 4/109 | most frequently GI disorders | nd/nd | nd/nd | 10/111 | 3/106 | nd/nd | nd/nd | nd/nd | nd/nd | 6/111 | 1/106 | nd/nd | nd/nd |
| Reasner et al., 2011 | 25/621 | 25/625 | nd | 24/621 | 7/625 | 103/621 | 75/625 | 16/621 | 18/625 | nd/nd | nd/nd | 39/621 | 35/625 | nd/nd | nd/nd |
| Rosenstock et al., 2010a | 2/50 | 2/51 | nd | 24/621 | nd/nd | 3\|50 | 3\|51 | nd/nd | nd/nd | nd/nd | nd/nd | 1\|50 | 2\|51 | nd/nd | nd/nd |
| Rosenstock et al., 2010b | 9/141 | 5/145 | most frequently diarrhea and hyperglycemia | 8/141 | 6/145 | 26/141 | 17/145 | 2/141 | 6/145 | 9/141 | 7/145 | 11/141 | 18/145 | 2/141 | 6/145 |
| Rosenstock et al.., 2006 | 3/154 | 5/159 | nd | nd/nd | nd/nd | 32/154 | 11/159 | 20/154 | 13/159 | nd/nd | nd/nd | 20/154 | 13/159 | nd/nd | nd/nd |
| Rosnstock et al., 2016 | 4/237 | 3/237 | nd | nd/nd | nd/nd | 3/237 | 3/237 | 7/237 | 1/137 | nd/nd | nd/nd | 7/237 | 1/237 | nd/nd | nd/nd |
| Russell-Jones et al., 2012 | 6/246 | 5/163 | most frequently nausea and vomiting | nd/nd | nd/nd | 31/246 | 6/163 | nd/nd | nd/nd | nd/nd | nd/nd | 17/246 | 7/163 | 8/246 | 3/163 |
| Schwartz et al., 2004 | 39/597 | 42/597 | most frequently diarrhea | nd/nd | nd/nd | 66/597 | 19/597 | nd/nd | nd/nd | nd/nd | nd/nd | 25/597 | 14/597 | nd/nd | nd/nd |
| Schwartz et al., 2006a | 2/178 | 7/174 | most frequently nausea and diarrhea | 9/178 | 4/174 | 25/178 | 25/174 | nd/nd | nd/nd | nd/nd | nd/nd | 17/178 | 19/174 | nd/nd | nd/nd |
| Schwartz et al., 2006b | 1/182 | 7/174 |  | 6/182 | 4/174 | 33/182 | 25/174 | nd/nd | nd/nd | nd/nd | nd/nd | 14/182 | 19/174 | nd/nd | nd/nd |
| Schwartz et al., 2006c | 0/172 | 7/174 |  | 4/172 | 4/174 | 27/172 | 25/174 | nd/nd | nd/nd | nd/nd | nd/nd | 14/172 | 19/174 | nd/nd | nd/nd |
| Scgweitzer et al., 2007 | 18/252 | 22/519 | most frequently GI disorders | 18/252 | 12/519 | 66/252 | 31/519 | 11/252 | 11/519 | 10/252 | 5/519 | 26/252 | 17/519 | 5/252 | 25/519 |
| Schweitzer et al., 2009 | 13/165 | 7/167 | most frequently GI disorders | nd/nd | nd/nd | 22/165 | 5/167 | nd/nd | nd/nd | nd/nd | nd/nd | 9/165 | 5/167 | nd/nd | nd/nd |
| Seino et al., 2012 | 0/100 | 0/92 | nd | nd/nd | nd/nd | 1/100 | 5\|92 | nd/nd | nd/nd | nd/nd | nd/nd | nd/nd | nd/nd | 2/100 | 1\|92 |
| Stewart et al., 2006 | 7/272 | 11/254 | only GI disorders | 1/272 | 0/254 | 49/272 | 20/254 | nd/nd | nd/nd | nd/nd | nd/nd | nd/nd | nd/nd | nd/nd | nd/nd |
| Sun et al., 2016 | 2\|54 | 3\|54 | most frequently GI disorders | nd/nd | nd/nd | 8\|54 | 0\|54 | nd/nd | nd/nd | 0/54 | paź.54 | nd/nd | nd/nd | nd/nd | nd/nd |
| Takeda et al., 2016 | 1/162 | 2/163 | nd | nd/nd | nd/nd | 5/161 | 4/161 | nd/nd | nd/nd | nd/nd | nd/nd | nd/nd | nd/nd | nd/nd | nd/nd |
| Takeshit et al., 2019 | 1\|42 | 0/42 | severe gastrointestinal symptom | nd/nd | nd/nd | 7\|42 | 0/42 | nd/nd | nd/nd | 2\|42 | 1\|42 | 1\|42 | 0/42 | 0/42 | 1\|42 |
| Umpierrez et al., 2014 | 12/268 | 8/270 | most frequently nausea | nd/nd | nd/nd | 37/268 | 21/270 | 13/268 | 20/270 | nd/nd | nd/nd | 43/268 | 31/270 | 3/268 | 13/270 |
| Weissman et al., 2005 | 37/384 | 28/382 | most frequently GI ­related and mild in nature | 43/384 | nd/nd | 63/384 | nd/nd | nd/nd | nd/nd | nd/nd | nd/nd | nd/nd | nd/nd | nd/nd | nd/nd |
| Williams -Herman et al., 2010a | 8/182 | 5/179 | nd | 7/182 | 9/179 | 14/182 | 8/179 | 0/182 | 1/179 | nd/nd | nd/nd | 6/182 | 2/179 | nd/nd | nd/nd |
| Williams -Herman et al., 2010b | 7/182 | 5/179 |  | 12/182 | 9/179 | 23/182 | 8/179 | 8/182 | 1/179 | nd/nd | nd/nd | 19/182 | 2/179 | nd/nd | nd/nd |
| Yan et al., 2011 | 3/287 | 6/283 | few GI disorders | nd/nd | nd/nd | 12/287 | 9/283 | nd/nd | nd/nd | nd/nd | nd/nd | nd/nd | nd/nd | nd/nd | nd/nd |
| Yoon et al., 2010 | 9/114 | 10/118 | nd | 10/114 | 10/118 | 10/114 | 4/118 | 10/114 | 10/114 | nd/nd | nd/nd | 10/114 | 10/118 | nd/nd | nd/nd |
| Yuan et al., 2012 | 0/26 | sty.33 | severe nausea | nd/nd | nd/nd | 3\|26 | 1\|33 | 2\|26 | 1\|33 | nd/nd | nd/nd | 2\|26 | 10\|33 | 1\|26 | 1\|33 |
| Yuxin et al., 2019a | nd/nd | nd/nd | nd | nd/nd | nd/nd | nd/nd | nd/nd | nd/nd | nd/nd | nd/nd | nd/nd | nd/nd | nd/nd | nd/nd | nd/nd |
| Yuxin et al., 2019b | nd/nd | nd/nd |  | 41/120 | 33/120 | 29/120 | 33/120 | 5/120 | 3/120 | 45/120 | 39/120 | 17/120 | 11/120 | nd/nd | nd/nd |
| Zack et al., 2014 | 0/28 | 0/28 | nd | nd/nd | nd/nd | 0/28 | 3\|28 | nd/nd | nd/nd | nd/nd | nd/nd | 0/28 | 4\|28 | 0/28 | 2\|28 |
| Zack et al., 2014 | 1\|25 | 0/25 | tooth abscess | nd/nd | nd/nd | 7\|25 | 1\|25 | nd/nd | nd/nd | nd/nd | nd/nd | 0/25 | 1/25 | nd/nd | nd/nd |
| List et al., 2009 | 1\|56 | 1\|54 | nd | nd/nd | nd/nd | 7\|56 | 4\|54 | nd/nd | nd/nd | nd/nd | nd/nd | 6\|56 | 3\|54 | nd/nd | nd/nd |
| ClinTrials et al., 2014 | 8/208 | 9/219 | nd | nd/nd | nd/nd | 20/208 | 6/219 | nd/nd | nd/nd | nd/nd | nd/nd | nd/nd | nd/nd | nd/nd | nd/nd |

Abbreviations: GI - gastrointestinal; MET - metformin; CTRL – control; nd – no data.

ESM Table S5. Risk of bias assessment.

| **Reference (name of first author) and publication year** | **Random sequence generation (selection bias)** | **Allocation concealment (selection bias)** | **Blinding of participants and personnel (performance bias)** | **Blinding of outcome assessment (detection bias) (patient-reported outcomes)** | **Blinding of outcome assessment (detection bias) (Mortality)** | **Incomplete outcome data addressed (attrition bias) (Short-term outcomes  (2-6 weeks))** | **Incomplete outcome data addressed (attrition bias) (Longer-term outcomes  (>6 weeks))** | **Selective reporting (reporting bias)** | **Other bias** | **No. Of Low assessments** |
| --- | --- | --- | --- | --- | --- | --- | --- | --- | --- | --- |
| Aggarwal et al., 2017 | ? | ? | L | L | ? | na | L | L | ? | 4 |
| Aschner et al., 2010 | L | ? | L | L | L | na | L | L | ? | 6 |
| Bailey et al., 2005 | L | ? | L | L | L | na | L | L | ? | 6 |
| Blonde et al., 2002 | ? | ? | L | L | L | na | L | L | ? | 5 |
| Ferrannini et al., 2013 | ? | ? | L | L | ? | na | L | L | ? | 4 |
| Bosi et al., 2009 | ? | ? | L | L | L | na | L | L | ? | 5 |
| DeFronzo et al., 2016 | L | L | L | L | L | na | L | L | ? | 7 |
| DeFronzo et al., 1995 | ? | ? | L | L | L | na | L | L | ? | 5 |
| Del Prato et al., 2002 | ? | ? | L | L | ? | na | L | L | ? | 4 |
| Derosa et al., 2017 | ? | ? | H | H | ? | na | L | L | ? | 2 |
| Dou et al., 2017 | L | ? | L | L | ? | na | L | L | ? | 5 |
| Henry et al., 2018 | L | ? | L | L | L | na | L | L | ? | 6 |
| Feinglos et al., 2005 | ? | ? | L | L | ? | na | L | L | ? | 4 |
| Fonseca et al., 2011 | L | ? | L | L | ? | na | L | L | ? | 5 |
| Fuangchan et al., 2011 | L | ? | L | L | ? | L | na | L | ? | 5 |
| Fujioka et al., 2005 | ? | ? | L | L | ? | na | L | L | ? | 4 |
| Fujioka et al., 2003 | ? | ? | L | L | ? | na | ? | ? | ? | 2 |
| Gao et al., 2008 | ? | ? | H | H | ? | na | L | L | ? | 2 |
| Gao et al., 2020 | ? | ? | H | H | ? | na | L | L | ? | 2 |
| Garber et al., 1997 | ? | ? | L | L | ? | na | L | L | ? | 4 |
| Garber et al., 2003 | L | L | L | L | ? | na | L | L | ? | 6 |
| Ghosh et al., 2014 | L | ? | H | H | ? | na | L | L | ? | 3 |
| Göke et al., 2008 | ? | ? | L | L | L | na | L | L | ? | 5 |
| Goldstein et al., 2003 | L | ? | L | L | ? | na | L | L | ? | 5 |
| Gottschalk et al., 2007 | ? | ? | H | H | ? | na | L | L | ? | 2 |
| Haak et al., 2012a | ? | ? | L | L | L | na | L | L | ? | 5 |
| Haak et al., 2012b | ? | ? | L | L | L | na | L | L | ? | 5 |
| Henry et al., 2012a | ? | L | L | L | L | na | L | L | ? | 6 |
| Henry et al., 2012b | ? | L | L | L | L | na | L | L | ? | 6 |
| Hemans et al., 2012 | ? | ? | L | L | L | na | L | L | ? | 5 |
| Horton et al., 2004 | ? | L | L | L | ? | na | H | ? | ? | 3 |
| Jadzinsky et al., 2009 | L | L | L | L | ? | na | L | L | ? | 6 |
| Ji et al., 2016a | ? | L | L | L | ? | na | L | L | ? | 5 |
| Ji et al., 2016b | ? | L | L | L | ? | na | L | L | ? | 5 |
| Ji et al., 2017 | ? | ? | H | H | L | na | L | L | ? | 3 |
| Ji et al., 2015 | ? | ? | L | L | ? | na | L | L | ? | 4 |
| Kahn et al., 2006 | L | L | L | L | L | na | L | L | ? | 7 |
| Kim et al., 2014 | ? | ? | H | H | L | na | L | L | ? | 3 |
| Lim et al., 2016 | L | ? | L | L | ? | na | L | L | ? | 4 |
| Ma et al., 2014 | ? | ? | ? | ? | ? | na | ? | L | ? | 1 |
| Moses et al., 1999 | ? | ? | L | L | ? | na | H | ? | ? | 2 |
| Nauck et al., 2012 | ? | ? | H | H | L | na | L | ? | ? | 2 |
| Nauck et al., 2008 | ? | L | L | L | L | na | L | L | ? | 6 |
| Neutel et al., 2013 | L | L | L | L | L | L | na | L | ? | 7 |
| Matthews et al., 2019 | L | L | L | L | L | na | L | L | ? | 7 |
| Oh et al., 2019 | ? | ? | L | L | ? | na | L | L | ? | 4 |
| Pavo et al., 2003 | ? | ? | L | L | ? | na | L | ? | ? | 3 |
| Perez et al., 2009 | ? | ? | L | L | ? | na | L | L | ? | 4 |
| Pratley et al., 2014a | ? | ? | L | L | ? | na | ? | L | ? | 3 |
| Pratley et al., 2014b | ? | ? | L | L | ? | na | ? | L | ? | 3 |
| Reasner et al., 2011 | ? | ? | L | L | L | na | L | L | ? | 5 |
| Rosenstock, Banarer et al., 2010 | ? | ? | L | L | L | na | L | L | ? | 5 |
| Rosenstock, Fonseca et al., 2010 | ? | ? | L | L | ? | na | L | L | ? | 4 |
| Rosenstock et al., 2016 | L | ? | L | L | ? | na | L | L | ? | 5 |
| Rosenstock et al., 2006 | H | H | L | L | ? | na | L | L | ? | 4 |
| Russell-Jones et al., 2012 | L | ? | L | ? | ? | na | L | L | ? | 4 |
| Schernthaner et al., 2004 | L | ? | L | L | L | na | L | L | ? | 6 |
| Schwartz et al., 2006 | ? | L | L | ? | L | na | L | L | ? | 5 |
| Schweizer et al., 2007 | ? | ? | L | L | L | na | L | L | ? | 4 |
| Schweizer et al., 2009 | ? | ? | L | L | L | na | L | L | ? | 5 |
| Seino et al., 2012 | ? | ? | L | L | L | na | L | L | ? | 5 |
| Stewartet al., 2006 | H | H | L | L | L | na | L | L | ? | 5 |
| Study of Dapagliflozin in Combination With Metformin XR to Initiate the Treatment of Type 2 Diabetes[cited 2020 Dec 7]; | ? | ? | L | L | L | na | L | L | ? | 5 |
| Sun et al., 2016 | ? | ? | ? | ? | ? | na | L | L | ? | 2 |
| Takeda et al., 2016 | L | L | L | L | L | na | L | L | ? | 7 |
| Takeshita et al., 2019 | L | ? | H | H | H | na | L | L | ? | 3 |
| Umpierrez et al., 2014 | L | L | L | ? | ? | na | L | L | ? | 5 |
| Weissman et al.,2005 | ? | ? | L | L | L | na | L | L | ? | 5 |
| Williams-Herman et al. 2010 | L | L | L | L | ? | na | L | L | ? | 6 |
| Yang et al., 2011 | ? | ? | L | L | L | na | L | L | ? | 5 |
| Yoon KH et al., 2011 | ? | ? | L | L | ? | na | L | L | ? | 4 |
| Yuan et al., 2012 | L | L | L | ? | ? | na | L | ? | ? | 4 |
| Yuxin et al., 2020 | ? | ? | ? | ? | ? | na | L | L | ? | 2 |
| Zack et al., 2015 | ? | ? | L | L | L | na | L | L | ? | 5 |

Abbreviations: L - low risk of bias; ? - some concerns; H - high risk of bias; na - not applicable

**References:**

[1] P. Aschner *et al.*, ‘Efficacy and safety of monotherapy of sitagliptin compared with metformin in patients with type 2 diabetes’, *Diabetes, Obes. \& Metab.*, vol. 12, no. 3, pp. 252–261, Mar. 2010, doi: 10.1111/j.1463-1326.2009.01187.x.

[2] L. Blonde, J. Rosenstock, A. D. Mooradian, B.-A. Piper, and D. Henry, ‘Glyburide/metformin combination product is safe and efficacious in patients with type 2 diabetes failing sulphonylurea therapy’, *Diabetes, Obes. Metab.*, vol. 4, no. 6, pp. 368–375, 2002, doi: 10.1046/j.1463-1326.2002.00229.x.

[3] E. Ferrannini *et al.*, ‘Long-Term Safety and Efficacy of Empagliflozin, Sitagliptin, and MetforminAn active-controlled, parallel-group, randomized, 78-week open-label extension study in patients with type 2 diabetes’, *Diabetes Care*, vol. 36, no. 12, pp. 4015–4021, Dec. 2013, doi: 10.2337/DC13-0663.

[4] E. Bosi, F. Dotta, Y. Jia, and M. Goodman, ‘Vildagliptin plus metformin combination therapy provides superior glycaemic control to individual monotherapy in treatment-naive patients with type 2 diabetes mellitus’, *Diabetes, Obes. Metab.*, vol. 11, no. 5, pp. 506–515, 2009, doi: 10.1111/j.1463-1326.2009.01040.x.

[5] R. A. Defronzo and A. M. Goodman, ‘Efficacy of metformin in patients with non-insulin-dependent diabetes mellitus’, *N. Engl. J. Med.*, vol. 333, no. 9, pp. 541–549, 1995, doi: 10.1056/NEJM199508313330902.

[6] S. Del Prato, D. W. Erkelens, and M. Leutenegger, ‘Six-month efficacy of benfluorex vs. placebo or metformin in diet-failed type 2 diabetic patients’, *Acta Diabetol.*, vol. 40, no. 1, pp. 20–27, Mar. 2003, doi: 10.1007/s005920300004.

[7] J. Dou *et al.*, ‘Efficacy and safety of saxagliptin in combination with metformin as initial therapy in Chinese patients with type 2 diabetes: Results from the START study, a multicentre, randomized, double-blind, active-controlled, phase 3 trial’, *Diabetes. Obes. Metab.*, vol. 20, no. 3, pp. 590–598, Mar. 2018, doi: 10.1111/DOM.13117.

[8] R. R. Henry *et al.*, ‘Improved glycemic control with minimal systemic metformin exposure: Effects of Metformin Delayed-Release (Metformin DR) targeting the lower bowel over 16 weeks in a randomized trial in subjects with type 2 diabetes’, *PLoS One*, vol. 13, no. 9, p. e0203946, Feb. 2018, doi: 10.1371/journal.pone.0203946.

[9] M. N. Feinglos, M. F. Saad, F. X. Pi-Sunyer, B. An, and O. Santiago, ‘Effects of liraglutide (NN2211), a long-acting GLP-1 analogue, on glycaemic control and bodyweight in subjects with Type 2 diabetes’, *Diabet. Med.*, vol. 22, no. 8, pp. 1016–1023, Aug. 2005, doi: 10.1111/J.1464-5491.2005.01567.X.

[10] A. Fuangchan *et al.*, ‘Hypoglycemic effect of bitter melon compared with metformin in newly diagnosed type 2 diabetes patients’, *J. Ethnopharmacol.*, vol. 134, no. 2, pp. 422–428, 2011, doi: 10.1016/j.jep.2010.12.045.

[11] K. Fujioka *et al.*, ‘Efficacy, dose-response relationship and safety of once-daily extended-release metformin (Glucophage XR) in type 2 diabetic patients with inadequate glycaemic control despite prior treatment with diet and exercise: results from two double-blind, placebo-controlled studies’, *Diabetes. Obes. Metab.*, vol. 7, no. 1, pp. 28–39, 2005, doi: 10.1111/J.1463-1326.2004.00369.X.

[12] A. J. Garber, T. G. Duncan, A. M. Goodman, D. J. Mills, and J. L. Rohlf, ‘Efficacy of Metformin in Type II Diabetes: Results of a Double-Blind, Placebo-controlled, Dose-Response Trial’, *Am. J. Med.*, vol. 103, no. 6, pp. 491–497, Dec. 1997, doi: 10.1016/S0002-9343(97)00254-4.

[13] A. J. Garber, D. S. Donovan Jr., P. Dandona, S. Bruce, and J.-S. Park, ‘Efficacy of glyburide/metformin tablets compared with initial monotherapy in type 2 diabetes’, *J. Clin. Endocrinol. Metab.*, vol. 88, no. 8, pp. 3598–3604, 2003, doi: 10.1210/jc.2002-021225.

[14] B. Göke *et al.*, ‘Efficacy and safety of vildagliptin monotherapy during 2-year treatment of drug-naïve patients with type 2 diabetes: Comparison with metformin’, *Horm. Metab. Res.*, vol. 40, no. 12, pp. 892–895, Dec. 2008, doi: 10.1055/S-0028-1082334/ID/11.

[15] B. J. Goldstein, M. Pans, and C. J. Rubin, ‘Multicenter, randomized, double-masked, parallel-group assessment of simultaneous glipizide/metformin as second-line pharmacologic treatment for patients with type 2 diabetes mellitus that is inadequately controlled by a sulfonylurea’, *Clin. Ther.*, vol. 25, no. 3, pp. 890–903, 2003, doi: 10.1016/S0149-2918(03)80112-1.

[16] T. Haak, T. Meinicke, R. Jones, S. Weber, M. V Eynatten, and H.-J. Woerle, ‘Initial combination of linagliptin and metformin improves glycaemic control in type 2 diabetes: {A} randomized, double-blind, placebo-controlled study’, *Diabetes, Obes. Metab.*, vol. 14, no. 6, pp. 565–574, 2012, doi: 10.1111/j.1463-1326.2012.01590.x.

[17] R. R. Henry, A. V Murray, M. H. Marmolejo, D. Hennicken, A. Ptaszynska, and J. F. List, ‘Dapagliflozin, metformin XR, or both: Initial pharmacotherapy for type 2 diabetes, a randomised controlled trial’, *Int. J. Clin. Pract.*, vol. 66, no. 5, pp. 446–456, 2012, doi: 10.1111/j.1742-1241.2012.02911.x.

[18] E. S. Horton, J. E. Foley, S. G. Shen, and M. A. Baron, ‘Efficacy and tolerability of initial combination therapy with nateglinide and metformin in treatment-naïve patients with type 2 diabetes’, *Curr. Med. Res. Opin.*, vol. 20, no. 6, pp. 883–889, 2004, doi: 10.1185/030079903125003881.

[19] M. Jadzinsky *et al.*, ‘Saxagliptin given in combination with metformin as initial therapy improves glycaemic control in patients with type 2 diabetes compared with either monotherapy: {A} randomized controlled trial’, *Diabetes, Obes. Metab.*, vol. 11, no. 6, pp. 611–622, 2009, doi: 10.1111/j.1463-1326.2009.01056.x.

[20] L. Ji *et al.*, ‘Randomized clinical trial of the safety and efficacy of sitagliptin and metformin co-administered to Chinese patients with type 2 diabetes mellitus’, *J. Diabetes Investig.*, vol. 7, no. 5, pp. 727–736, Sep. 2016, doi: 10.1111/JDI.12511.

[21] S. E. Kahn *et al.*, ‘Glycemic durability of rosiglitazone, metformin, or glyburide monotherapy’, *N. Engl. J. Med.*, vol. 355, no. 23, pp. 2427–2443, 2006, doi: 10.1056/NEJMoa066224.

[22] S. Lim *et al.*, ‘Efficacy and safety of initial combination therapy with gemigliptin and metformin compared with monotherapy with either drug in patients with type 2 diabetes: A double‐blind randomized controlled trial (INICOM study)’, *Diabetes. Obes. Metab.*, vol. 19, no. 1, p. 87, Jan. 2017, doi: 10.1111/DOM.12787.

[23] ‘Comparison of Metformin and Repaglinide Monotherapy in the Treatment of New Onset Type 2 Diabetes Mellitus in China - Full Text View - ClinicalTrials.gov’. https://clinicaltrials.gov/ct2/show/NCT02040246 (accessed May 02, 2022).

[24] R. Moses *et al.*, ‘Effect of repaglinide addition to metformin monotherapy on glycemic control in patients with type 2 diabetes’, *Diabetes Care*, vol. 22, no. 1, pp. 119–124, 1999, doi: 10.2337/diacare.22.1.119.

[25] I. Pavo *et al.*, ‘Effect of pioglitazone compared with metformin on glycemic control and indicators of insulin sensitivity in recently diagnosed patients with type 2 diabetes’, *J. Clin. Endocrinol. Metab.*, vol. 88, no. 4, pp. 1637–1645, 2003, doi: 10.1210/jc.2002-021786.

[26] A. Perez, Z. Zhao, R. Jacks, and R. Spanheimer, ‘Efficacy and safety of pioglitazone/metformin fixed-dose combination therapy compared with pioglitazone and metformin monotherapy in treating patients with T2DM’, *Curr. Med. Res. Opin.*, vol. 25, no. 12, pp. 2915–2923, Dec. 2009, doi: 10.1185/03007990903350011.

[27] R. E. Pratley, P. Fleck, and C. Wilson, ‘Efficacy and safety of initial combination therapy with alogliptin plus metformin versus either as monotherapy in drug-naïve patients with type 2 diabetes: a randomized, double-blind, 6-month study’, *Diabetes, Obes. Metab.*, vol. 16, no. 7, pp. 613–621, Jul. 2014, doi: 10.1111/DOM.12258.

[28] J. Rosenstock, J. Rood, A. Cobitz, N. Biswas, H. Chou, and A. Garber, ‘Initial treatment with rosiglitazone/metformin fixed-dose combination therapy compared with monotherapy with either rosiglitazone or metformin in patients with uncontrolled type 2 diabetes’, *Diabetes, Obes. \& Metab.*, vol. 8, no. 6, pp. 650–660, Nov. 2006, doi: 10.1111/j.1463-1326.2006.00659.x.

[29] J. Rosenstock *et al.*, ‘Initial Combination Therapy With Canagliflozin Plus Metformin Versus Each Component as Monotherapy for Drug-Naïve Type 2 Diabetes’, *Diabetes Care*, vol. 39, no. 3, pp. 353–362, Dec. 2016, doi: 10.2337/dc15-1736.

[30] D. Russell-Jones *et al.*, ‘Efficacy and safety of exenatide once weekly versus metformin, pioglitazone, and sitagliptin used as monotherapy in drug-naive patients with type 2 diabetes (DURATION-4): a 26-week double-blind study’, *Diabetes Care*, vol. 35, no. 2, pp. 252–258, 2012, doi: 10.2337/dc11-1107.

[31] G. Schernthaner, D. R. Matthews, B. Charbonnel, M. Hanefeld, and P. Brunetti, ‘Efficacy and Safety of Pioglitazone Versus Metformin in Patients with Type 2 Diabetes Mellitus: A Double-Blind, Randomized Trial’, *J. Clin. Endocrinol. Metab.*, vol. 89, no. 12, pp. 6068–6076, Nov. 2004, doi: 10.1210/jc.2003-030861.

[32] A. Schweizer, A. Couturier, J. E. Foley, and S. Dejager, ‘Comparison between vildagliptin and metformin to sustain reductions in HbA(1c) over 1 year in drug-naïve patients with Type 2 diabetes’, *Diabet. Med.*, vol. 24, no. 9, pp. 955–961, Sep. 2007, doi: 10.1111/J.1464-5491.2007.02191.X.

[33] A. Schweizer, S. Dejager, and E. Bosi, ‘Comparison of vildagliptin and metformin monotherapy in elderly patients with type 2 diabetes: a 24-week, double-blind, randomized trial’, *Diabetes, Obes. \& Metab.*, vol. 11, no. 8, pp. 804–812, Aug. 2009, doi: 10.1111/j.1463-1326.2009.01051.x.

[34] W. Sun, C. Zeng, L. Liao, J. Chen, and Y. Wang, ‘Comparison of acarbose and metformin therapy in newly diagnosed type 2 diabetic patients with overweight and/or obesity’, *Curr. Med. Res. Opin.*, vol. 32, no. 8, pp. 1389–1396, 2016, doi: 10.1080/03007995.2016.1176013.

[35] ‘Efficacy and Safety of Alogliptin and Metformin Fixed-Dose Combination in Participants With Type 2 Diabetes - Full Text View - ClinicalTrials.gov’. https://clinicaltrials.gov/ct2/show/NCT01890122 (accessed May 02, 2022).

[36] Y. Takeshita *et al.*, ‘Effects of metformin and alogliptin on body composition in people with type 2 diabetes’, *J. Diabetes Investig.*, vol. 10, no. 3, pp. 723–730, 2019, doi: 10.1111/jdi.12920.

[37] G. Umpierrez, S. Tofé Povedano, F. Pérez Manghi, L. Shurzinske, and V. Pechtner, ‘Efficacy and safety of dulaglutide monotherapy versus metformin in type 2 diabetes in a randomized controlled trial (AWARD-3)’, *Diabetes Care*, vol. 37, no. 8, pp. 2168–2176, 2014, doi: 10.2337/dc13-2759.

[38] D. Williams-Herman *et al.*, ‘Efficacy and safety of sitagliptin and metformin as initial combination therapy and as monotherapy over 2 years in patients with type 2 diabetes’, *Diabetes, Obes. Metab.*, vol. 12, no. 5, pp. 442–451, May 2010, doi: 10.1111/J.1463-1326.2010.01204.X.

[39] K. H. Yoon *et al.*, ‘Comparison of the efficacy of glimepiride, metformin, and rosiglitazone monotherapy in korean drug-naïve type 2 diabetic patients: the practical evidence of antidiabetic monotherapy study’, *Diabetes \& Metab. J.*, vol. 35, no. 1, pp. 26–33, Feb. 2011, doi: 10.4093/dmj.2011.35.1.26.

[40] G.-H. Yuan, W.-L. Song, Y.-Y. Huang, X.-H. Guo, and Y. Gao, ‘Efficacy and tolerability of exenatide monotherapy in obese patients with newly diagnosed type 2 diabetes: {A} randomized, 26 weeks metformin-controlled, parallel-group study’, *Chin. Med. J. (Engl).*, vol. 125, no. 15, pp. 2677–2681, 2012, doi: 10.3760/cma.j.issn.0366-6999.2012.15.006.

[41] J. F. List, V. Woo, E. Morales, W. Tang, and F. T. Fiedorek, ‘Sodium-Glucose Cotransport Inhibition With Dapagliflozin in Type 2 Diabetes’, *Diabetes Care*, vol. 32, no. 4, pp. 650–657, Feb. 2009, doi: 10.2337/dc08-1863.

[42] ‘Study of Dapagliflozin in Combination With Metformin XR to Initiate the Treatment of Type 2 Diabetes - Full Text View - ClinicalTrials.gov’. https://clinicaltrials.gov/ct2/show/NCT00859898 (accessed May 02, 2022).

[43] N. Aggarwal *et al.*, ‘Metformin extended-release versus immediate-release: An international, randomized, double-blind, head-to-head trial in pharmacotherapy-naïve patients with type 2 diabetes.’, *Diabetes. Obes. Metab.*, vol. 20, no. 2, pp. 463–467, Feb. 2018, doi: 10.1111/dom.13104.

[44] R. A. DeFronzo *et al.*, ‘Once-daily delayed-release metformin lowers plasma glucose and enhances fasting and postprandial {GLP}-1 and {PYY}: results from two randomised trials’, *Diabetologia*, vol. 59, no. 8, pp. 1645–1654, 2016, doi: 10.1007/s00125-016-3992-6.

[45] G. Derosa, A. D’Angelo, D. Romano, and P. Maffioli, ‘Effects of metformin extended release compared to immediate release formula on glycemic control and glycemic variability in patients with type 2 diabetes’, *Drug Des. Devel. Ther.*, vol. 11, pp. 1481–1488, May 2017, doi: 10.2147/DDDT.S131670.

[46] K. Fujioka, M. Pans, and S. Joyal, ‘Glycemic control in patients with type 2 diabetes mellitus switched from twice-daily immediate-release metformin to a once-daily extended-release formulation’, *Clin. Ther.*, vol. 25, no. 2, pp. 515–529, 2003, doi: 10.1016/S0149-2918(03)80093-0.

[47] H. Gao *et al.*, ‘The metabolic effects of once daily extended-release metformin in patients with type 2 diabetes: {A} multicentre study’, *Int. J. Clin. Pract.*, vol. 62, no. 5, pp. 695–700, 2008, doi: 10.1111/j.1742-1241.2008.01733.x.

[48] L. Ji *et al.*, ‘Comparative effectiveness of metformin monotherapy in extended release and immediate release formulations for the treatment of type 2 diabetes in treatment-naïve Chinese patients: Analysis of results from the CONSENT trial’, *Diabetes. Obes. Metab.*, vol. 20, no. 4, pp. 1006–1013, Apr. 2018, doi: 10.1111/DOM.13190.

[49] S. Schwartz, V. Fonseca, B. Berner, M. Cramer, Y.-K. Chiang, and A. Lewin, ‘Efficacy, tolerability, and safety of a novel once-daily extended-release metformin in patients with type 2 diabetes’, *Diabetes Care*, vol. 29, no. 4, pp. 759–764, 2006, doi: 10.2337/diacare.29.04.06.dc05-1967.

[50] C. J. Bailey *et al.*, ‘Rosiglitazone/metformin fixed-dose combination compared with uptitrated metformin alone in type 2 diabetes mellitus: a 24-week, multicenter, randomized, double-blind, parallel-group study’, *Clin. Ther.*, vol. 27, no. 10, pp. 1548–1561, Oct. 2005, doi: 10.1016/j.clinthera.2005.10.012.

[51] V. Fonseca, T. Zhu, C. Karyekar, and B. Hirshberg, ‘Adding saxagliptin to extended-release metformin vs. uptitrating metformin dosage’, *Diabetes, Obes. Metab.*, vol. 14, no. 4, pp. 365–371, Apr. 2012, doi: 10.1111/j.1463-1326.2011.01553.x.

[52] Y. Gao, L. Gao, Y. Peng, J. Long, and M. Yang, ‘Therapeutic effects of the combination of linagliptin and metformin on the treatment of elderly type 2 diabetes mellitus and influences on serum uric acid, insulin resistance and insulin Â cell functions’, *Acta Medica Mediterr.*, vol. 36, no. 1, pp. 421–425, 2020, doi: 10.19193/0393-6384_2020_1_66.

[53] A. Ghosh, N. Sengupta, P. Sahana, D. Giri, P. Sengupta, and N. Das, ‘Efficacy and safety of add on therapy of bromocriptine with metformin in Indian patients with type 2 diabetes mellitus: A randomized open labeled phase IV clinical trial’, *Indian J. Pharmacol.*, vol. 46, no. 1, p. 24, Jan. 2014, doi: 10.4103/0253-7613.125160.

[54] M. Gottschalk, T. Danne, A. Vlajnic, and J. F. Cara, ‘Glimepiride versus metformin as monotherapy in pediatric patients with type 2 diabetes: {A} randomized, single-blind comparative study’, *Diabetes Care*, vol. 30, no. 4, pp. 790–794, 2007, doi: 10.2337/dc06-1554.

[55] M. P. Hermans *et al.*, ‘Effects of saxagliptin added to sub-maximal doses of metformin compared with uptitration of metformin in type 2 diabetes: the PROMPT study’, *Curr. Med. Res. Opin.*, vol. 28, no. 10, pp. 1635–1645, Oct. 2012, doi: 10.1185/03007995.2012.735646.

[56] L. Ji *et al.*, ‘Efficacy and safety of linagliptin co-administered with low-dose metformin once daily versus high-dose metformin twice daily in treatment-naïve patients with type 2 diabetes: a double-blind randomized trial’, *Adv. Ther.*, vol. 32, no. 3, pp. 201–215, Mar. 2015, doi: 10.1007/s12325-015-0195-3.

[57] H.-S. Kim *et al.*, ‘Efficacy of glimepiride/metformin fixed-dose combination vs metformin uptitration in type 2 diabetic patients inadequately controlled on low-dose metformin monotherapy: A randomized, open label, parallel group, multicenter study in Korea’, *J. Diabetes Investig.*, vol. 5, no. 6, pp. 701–708, 2014, doi: 10.1111/jdi.12201.

[58] M. Nauck *et al.*, ‘Long-term efficacy and safety comparison of liraglutide, glimepiride and placebo, all in combination with metformin in type 2 diabetes: 2-year results from the LEAD-2 study’, *Diabetes. Obes. Metab.*, vol. 15, no. 3, pp. 204–212, 2013, doi: 10.1111/DOM.12012.

[59] M. A. Nauck, G. C. Ellis, P. R. Fleck, C. A. Wilson, and Q. Mekki, ‘Efficacy and safety of adding the dipeptidyl peptidase-4 inhibitor alogliptin to metformin therapy in patients with type 2 diabetes inadequately controlled with metformin monotherapy: {A} multicentre, randomised, double-blind, placebo-controlled study’, *Int. J. Clin. Pract.*, vol. 63, no. 1, pp. 46–55, 2009, doi: 10.1111/j.1742-1241.2008.01933.x.

[60] J. M. Neutel, C. Zhao, and C. S. Karyekar, ‘Adding Saxagliptin to Metformin Extended Release (XR) or Uptitration of Metformin XR: Efficacy on Daily Glucose Measures’, *Diabetes Ther.*, vol. 4, no. 2, pp. 269–283, Dec. 2013, doi: 10.1007/S13300-013-0028-9.

[61] D. R. Matthews, P. M. Paldánius, P. Proot, Y. T. Chiang, M. Stumvoll, and S. Del Prato, ‘Glycaemic durability of an early combination therapy with vildagliptin and metformin versus sequential metformin monotherapy in newly diagnosed type 2 diabetes (VERIFY): a 5-year, multicentre, randomised, double-blind trial’, *Lancet*, vol. 394, no. 10208, pp. 1519–1529, Oct. 2019, doi: 10.1016/S0140-6736(19)32131-2.

[62] T. J. Oh *et al.*, ‘Efficacy and safety of voglibose plus metformin in patients with type 2 diabetes mellitus: {A} randomized controlled trial’, *Diabetes Metab. J.*, vol. 43, no. 3, pp. 276–286, 2019, doi: 10.4093/dmj.2018.0051.

[63] C. Reasner *et al.*, ‘The effect of initial therapy with the fixed-dose combination of sitagliptin and metformin compared with metformin monotherapy in patients with type 2 diabetes mellitus’, *Diabetes, Obes. \& Metab.*, vol. 13, no. 7, pp. 644–652, Jul. 2011, doi: 10.1111/j.1463-1326.2011.01390.x.

[64] J. Rosenstock *et al.*, ‘The 11-β-hydroxysteroid dehydrogenase type 1 inhibitor {INCB13739} improves hyperglycemia in patients with type 2 diabetes inadequately controlled by metformin monotherapy’, *Diabetes Care*, vol. 33, no. 7, pp. 1516–1522, 2010, doi: 10.2337/dc09-2315.

[65] J. Rosenstock *et al.*, ‘Initial Combination Therapy with Metformin and Colesevelam for Achievement of Glycemic and Lipid Goals Min Early Type 2 Diabetes’, *Endocr. Pract.*, vol. 16, no. 4, pp. 629–640, Jan. 2010, doi: 10.4158/EP10130.OR.

[66] Y. Seino, Y. Miyata, S. Hiroi, M. Hirayama, and K. Kaku, ‘Efficacy and safety of alogliptin added to metformin in {Japanese} patients with type 2 diabetes: {A} randomized, double-blind, placebo-controlled trial with an open-label, long-term extension study’, *Diabetes, Obes. Metab.*, vol. 14, no. 10, pp. 927–936, 2012, doi: 10.1111/j.1463-1326.2012.01620.x.

[67] M. W. Stewart *et al.*, ‘Effect of metformin plus roziglitazone compared with metformin alone on glycaemic control in well-controlled {Type} 2 diabetes’, *Diabet. Med. A J. Br. Diabet. Assoc.*, vol. 23, no. 10, pp. 1069–1078, Oct. 2006, doi: 10.1111/j.1464-5491.2006.01942.x.

[68] P. Weissman *et al.*, ‘Effects of rosiglitazone added to submaximal doses of metformin compared with dose escalation of metformin in type 2 diabetes: the EMPIRE Study’, *Curr. Med. Res. Opin.*, vol. 21, no. 12, pp. 2029–2035, Dec. 2005, doi: 10.1185/030079905X74844.

[69] W. Yang, C. Y. Pan, C. Tou, J. Zhao, and I. Gause-Nilsson, ‘Efficacy and safety of saxagliptin added to metformin in Asian people with type 2 diabetes mellitus: a randomized controlled trial’, *Diabetes Res. Clin. Pract.*, vol. 94, no. 2, pp. 217–224, Nov. 2011, doi: 10.1016/J.DIABRES.2011.07.035.

[70] H. Yuxin *et al.*, ‘Comparison of gastrointestinal adverse events with different doses of metformin in the treatment of elderly people with type 2 diabetes’, *J. Clin. Pharm. Ther.*, vol. 45, no. 3, pp. 470–476, Jun. 2020, doi: 10.1111/JCPT.13087.

[71] J. Zack *et al.*, ‘Pharmacokinetic drug–drug interaction study of ranolazine and metformin in subjects with type 2 diabetes mellitus’, *Clin. Pharmacol. Drug Dev.*, vol. 4, no. 2, pp. 121–129, Mar. 2015, doi: 10.1002/CPDD.174.
